# Particle size analysis of pristine food-grade titanium dioxide and E 171 in confectionery products: Interlaboratory testing of a single-particle inductively coupled plasma mass spectrometry screening method and confirmation with transmission electron microscopy

**DOI:** 10.1016/j.foodcont.2020.107550

**Published:** 2021-02

**Authors:** Otmar Geiss, Ivana Bianchi, Chiara Senaldi, Guillaume Bucher, Eveline Verleysen, Nadia Waegeneers, Frédéric Brassinne, Jan Mast, Katrin Loeschner, Janja Vidmar, Federica Aureli, Francesco Cubadda, Andrea Raggi, Francesca Iacoponi, Ruud Peters, Anna Undas, Alexandra Müller, Ann-Katrin Meinhardt, Elke Walz, Volker Gräf, Josefa Barrero-Moreno

**Affiliations:** a European Commission, Joint Research Centre (JRC), Ispra, Italy; b Service Commun des Laboratoires (SCL), 3 Avenue Dr Albert Schweitzer, 33600, Pessac, France; c Sciensano, Trace Elements and Nanomaterials, Uccle/Tervuren, Belgium; d Division for Food Technology, National Food Institute, Technical University of Denmark, Kemitorvet 201, DK-2800 Kgs. Lyngby, Denmark; e Istituto Superiore di Sanità (ISS), National Institute of Health, Rome, Italy; f Wageningen Food Safety Research (WFSR), Wageningen University & Research, Business Unit Contaminants & Toxins, Akkermaalsbos 2, 6708, WB Wageningen, the Netherlands; g Max Rubner-Institut (MRI), Federal Research Institute of Nutrition and Food, Department of Food Technology and Bioprocess Engineering, Haid-und-Neu-Straße 9, 76131, Karlsruhe, Germany

**Keywords:** Single-particle ICP-MS, Food-grade titanium dioxide, E 171, Confectionery, Validation

## Abstract

Titanium dioxide is a white colourant authorised as food additive E 171 in the EU, where it is used in a range of alimentary products. As these materials may contain a fraction of particulates with sizes below 100 nm and current EU regulation requires specific labelling of food ingredient to indicate the presence of engineered nanomaterials there is now a need for standardised and validated methods to appropriately size and quantify (nano)particles in food matrices.

A single-particle inductively coupled plasma mass spectrometry (spICP-MS) screening method for the determination of the size distribution and concentration of titanium dioxide particles in sugar-coated confectionery and pristine food-grade titanium dioxide was developed. Special emphasis was placed on the sample preparation procedure, crucial to reproducibly disperse the particles before analysis. The transferability of this method was tested in an interlaboratory comparison study among seven experienced European food control and food research laboratories equipped with various ICP-MS instruments and using different software packages. The assessed measurands included the particle mean diameter, the most frequent diameter, the percentage of particles (in number) with a diameter below 100 nm, the particles' number concentration and a number of cumulative particle size distribution parameters (D0, D10, D50, D99.5, D99.8 and D100). The evaluated method's performance characteristics were, the within-laboratory precision, expressed as the relative repeatability standard deviation (RSDr), and the between-laboratory precision, expressed as the relative reproducibility standard deviation (RSDR). Transmission electron microscopy (TEM) was used as a confirmatory technique and served as the basis for bias estimation.

The optimisation of the sample preparation step showed that when this protocol was applied to the relatively simple sample food matrices used in this study, bath sonication turned out to be sufficient to reach the highest, achievable degree of dispersed constituent particles. For the pristine material, probe sonication was required. Repeatability and reproducibility were below 10% and 25% respectively for most measurands except for the lower (D0) and the upper (D100) bound of the particle size distribution and the particle number concentration. The broader distribution of the lower and the upper bounds could be attributed to instrument-specific settings/setups (e.g. the timing parameters, the transport efficiency, type of mass-spectrometer) and software-specific data treatment algorithms. Differences in the upper bound were identified as being due to the non-harmonised application of the upper counting limit. Reporting D99.5 or D99.8 instead of the effectively largest particle diameter (D100) excluded isolated large particles and considerably improved the reproducibility. The particle number-concentration was found to be influenced by small differences in the sample preparation procedure. The comparison of these results with those obtained using electron microscopy showed that the mean and median particle diameter was, in all cases, higher when using spICP-MS. The main reason for this was the higher size detection limit for spICP-MS plus the fact that some of the analysed particles remained agglomerated/aggregated after sonication.

Single particle ICP-MS is a powerful screening technique, which in many cases provides sufficient evidence to confirm the need to label a food product as containing (engineered) titanium dioxide nanomaterial according to the current EU regulatory requirements. The overall positive outcome of the method performance evaluation and the current lack of alternative standardised procedures, would indicate this method as being a promising candidate for a full validation study.

## Highlights

• Standardised methods for sizing of E171 particles in food matrices are unavailable.
• Screening method based on spICP-MS is proposed.
• Analytical performance parameters and transferability tested in ILC.

## 1 Introduction

Titanium dioxide (E 171) is an authorised food additive in the European Union (EU) (European Commission, 2008). It is used in many food products, such as chewing gum, and in confectionery, such as sugar-coated candies, for its colouring and opacifying properties (Chen et al., 2013; Dudefoi et al., 2017; Weir, Westerhoff, Fabricius, Hristovski, & von Goetz, 2012). As a food additive, within the EU, it must be labelled according to the provisions of Regulation (EU) No. 1169/2011 (European Commission, 2011a). Furthermore, if it is also present in the form of nanoparticles (i.e., complying with the definition of an engineered nanomaterial in Regulation (EU) No. 2015/2283), it must be indicated in the list of ingredients by the addition of the suffix ‘nano’ in brackets. The European Food Safety Authority (EFSA) has recently published a scientific opinion on the proposed amendment to the EU specifications for titanium dioxide (E 171) with respect to the inclusion of additional parameters related to its particle size distribution (Younes et al., 2019). Should the proposed amendment be adopted, EU specifications might require that the median minimum external dimension of the constituent particles needs to be above 100 nm (>100 nm), i.e., that the material contains less than 50% (<50%) of constituent particles (by number) with a minimum external dimension of below 100 nm (<100 nm), as determined with electron microscopy (EM).

Methods for regulatory compliance testing that are fit for this purpose are required and need to be nanoparticle and element specific (Hardy et al., 2018) while also being capable of determining number-based particle size distributions. Electron microscopy combined with energy dispersive X-ray spectroscopy (EDX) and single-particle ICP-MS (spICP-MS) meet these requirements.

Electron microscopy is a well-recognized tool for nanomaterial characterisation, recommended by the EFSA for the size measurement of nanomaterials in food (Hardy et al., 2018). Transmission electron microscopy (TEM) has been proven to also be a suitable method for titanium dioxide particle analysis in a number of matrices (Dudefoi et al., 2018; Geiss et al., 2019; Lu et al., 2018; Verleysen, De Temmerman, Van Doren, Abi Daoud Francisco, & Mast, 2014; Weir et al., 2012). It is currently the only analytical technique that can be expected to quantify the constituent particle size-distribution over the full size-range. On the other hand, spICP-MS has been proven to be a powerful tool to directly quantify particle size, concentration, and size distribution. In spICP-MS, an elemental mass spectrometer detects the non-continuous pulse signals that are generated by each nanoparticle entering the plasma (International Organization for Standardization, 2017a; Pace et al., 2012) and has been used in numerous studies to determine the particle size distribution of food-grade titanium dioxide (Bucher & Auger, 2019; Candás-Zapico, Kutscher, Montes-Bayón, & Bettmer, 2018; Dan, Shi, Stephan, & Liang, 2015; Donovan et al., 2016; Geiss et al., 2019; Peters et al., 2014; Verleysen et al., 2020; Vidmar, Loeschner, & Larios, 2019). These studies however have highlighted certain limitations of the technique as compared to TEM. For example, the size detection limit for titanium dioxide which is approximately 30–40 nm, depending on the type of instrument, and the inability to distinguish between constituent and agglomerated/aggregated particles. These limitations permit spICP-MS to be used primarily as a screening technique while TEM can operate as a confirmatory technique.

In 2018 the Joint Research Centre (JRC) of the European Commission organised a meeting with representatives of EU-Member State (MS) food control laboratories in charge of the enforcement of the legislative framework related to nanomaterials in food. The aim of that meeting was to discuss the challenges and needs faced by the control laboratories for enforcement of the current legislative framework in relation to the ingredient labelling. One of the challenges both food control and food research laboratories (but also other economic operators) are facing when testing the food additive E 171 is the lack of official, validated analytical methods. It was therefore agreed to join forces towards progressing on method development/validation. This study proposes a screening method specifically for the sizing and quantification of (engineered) nanoparticles in selected types of confectionery. Its analytical performance and transferability in a variety of laboratories were successfully tested in an interlaboratory comparison (ILC) study in seven experienced European food control and food research laboratories. No such study specifically for the determination of food-grade titanium dioxide in food is currently available. The method proposed in this study and the results obtained during the ILC, may serve as preliminary step towards full validation of the method.

## 2 Materials and methods

### 2.1 Test samples

This study specifically focused on confectionery products, which contained titanium dioxide (E 171) in the outer sugar shell only, following a relatively straightforward sample preparation procedure while covering many such products on the market. The products chosen were a) button-shaped chocolate candies covered with a hard layer of sugar and food colouring and b) white chewing gum dragées (coated pellets). To reduce any potential variability due to different coloration, only yellow candies, purchased directly from the producer, were analysed. The chewing gum dragées were purchased through an online shop shipping traditional Dutch products abroad. Pictures of both confectionery samples are provided in the supplementary material (SM1) and the complete lists of ingredients of both are shown in Table 1. In addition to the commercially available confectionery samples, a pristine food-grade anatase titanium dioxide material (powder) was also tested. The chosen pristine material was a well-characterised material provided by Sciensano in Belgium, representing anatase E 171 food additives.

**Table 1:** Ingredient lists of confectionery samples.

Confectionery	List of ingredients
Button-shaped Candies	Sugar, cocoa paste, skimmed milk powder, cocoa butter, lactose, milk fats, palm fat, glucose syrup, starch, shea butter, dyes (E 100, E 120, E 132, E 133, E 150a, E 150c, E 150d, E 153, E 160a, E 160e, E 162, E 163, E 171, E 172), dextrins, coating agents (beeswax, carnauba wax), emulsifiers (soy lecithin, E445), coconut oil, salt, aromas
Chewing Gum Dragées	Sweeteners (sorbitol, isomalt, maltitol syrup, maltitol, aspartame, acesulfame K), gum base, bulking agent (E 170), flavourings, liquorice extract, thickener (E 414), dye (E 171), emulsifier (sunflower lecithin), coating agent (E 903), antioxidant (E 321)

### 2.2 Homogeneity testing

Sample homogeneity studies were conducted on the food samples using spICP-MS and on the pristine titanium dioxide sample using transmission electron microscopy (TEM). The procedures and results can be found in the supplementary material (SM2).

### 2.3 Sample preparation and optimisation of sonication conditions

Before the actual analytical measurement, it is important to reproducibly disperse the particles in such a way that the resulting dispersions are stable and contain only, or mainly, single (primary) constituent particles (NanoDefine, 2016). More specifically, the sample sonication conditions play an important role in this regard and could alter the agglomeration state of the particles and consequently have an impact on the particle size distribution for those techniques that are unable to selectively measure constituent particles. Therefore, the minimum sonication conditions required to achieve the lowest possible agglomeration state were investigated in this study for all three sample materials (chewing gum, candies and the pristine E 171). Sample extracts/suspensions were submitted to four different treatments: no sonication, bath sonication (Starsonic 35, PBI International, Milan, Italy) and probe sonication (VibraCell VCX-130, 3 mm tip, Sonics & Materials Inc, Newton, CT, USA) at 5 kJ and 10 kJ of delivered energy. Three replicates and a blank were prepared for each sonication condition and for each sample material resulting in a total number of 48 samples. A scheme of all analysed samples can be found in the supplementary material (SM3). The effective delivered acoustic power of the sonication probe and bath was determined following the approach described by Taurozzi and co-workers (Taurozzi, Hackley, & Wiesner, 2011) and was 18 W and 2 W, respectively. After sonication the agglomeration state of each suspension was assessed with spICP-MS, centrifugal liquid sedimentation (CLS) and TEM. Since the sonication tips were made of a titanium alloy which, after a certain time of operation, tend to erode (Betts, Johnson, Rygiewicz, King, & Andersen, 2013; Hackley & Wiesner, 2010) a new tip was purchased for this set of measurements to minimise any possible contamination during sonication. No visual degradation was observed over the entire operational period and the absence of particles in the procedural blanks confirmed this.

Sample preparation was largely based on a procedure described by Bucher and Auger (Bucher & Auger, 2019). A defined number of confectionery units (6 candies and 3 chewing gum dragées) and a defined amount of pristine E 171 powder (Table 3) were weighed in 50 mL disposable plastic tubes. The titanium dioxide contained in the confectionery's coating was dissolved and dispersed by adding 25 mL of ultrapure water and manually shaking until only the chocolate core of the candies and the dark-grey gum-base of the chewing gum became visible. The chocolate core and the gum base were then respectively removed from the suspension and ultrapure water was added up to a volume of 35 mL. The tube containing pristine titanium dioxide was directly brought to the volumes detailed in Table 3. All sample suspensions were then vortex-stirred for 30 s. For each of the four sonication conditions to be tested, an aliquot was taken from the 35 mL suspension and transferred into a 15 mL plastic tube (5 mL for suspensions that were not sonicated and 10 mL for suspensions that were sonicated at various energies). The aliquots were then bath and probe sonicated under the conditions detailed in Table 2. To avoid overheating, the samples were immersed in an ice bath during probe sonication.

**Table 2:** Sonication conditions applied to sample extracts.

	Delivered acoustic power^a^[W]	Sonication time [s/min]	Delivered energy [J]	Suspension volume [mL]	Energy density [J mL^−1^]
No sonication	0	0	0	5	0
Bath sonication	2	600/10	1200	10	120
Probe sonication 90% amplitude	18	300/5	5400	10	540
Probe sonication 90% amplitude	18	540/9	9720	10	972

a Determined according to Taurozzi et al. (Taurozzi et al., 2011).

**Table 3:** Summary of sample preparation steps.

	Pristine E 171 (TEM)	Pristine E 171 (spICP-MS & CLS)	Button-shaped candies	Chewing gum
Preparation of suspension				
Amount of sample	88 mg	40 mg	6 units	3 units
Volume of ultrapure water added [mL]	35	40	35	35
Approximate concentration of TiO_2_ [mg mL^−1^] a	2.5	1.0	0.17	0.16
Sonication Step – Volume of water added to the sample and sonication time				
No Sonication	5 mL/0 min	5 mL/0 min	5 mL/0 min	5 mL/0 min
Bath Sonication	10 mL/10 min	10 mL/10 min	10 mL/10 min	10 mL/10 min
Probe Sonication 5 kJ	10 mL/5 min	10 mL/5 min	10 mL/5 min	10 mL/5 min
Probe Sonication 10 kJ	10 mL/9 min	10 mL/9 min	10 mL/9 min	10 mL/9 min
pH verification				
Dilutions				
CLS	n/a	1:30	1:30	1:30
spICP-MS	n/a	1:60,000^b^	1:60,000^b^	1:60,000^b^
TEM	Undiluted.c = 2.5 mg mL^−1^		Suspensions required cleaning to remove colorants and sugar before application to a TEM grid. The complete procedure is described in section 2.4.2.	

a The amount of titanium dioxide in one unit of button-shaped candy and one unit of chewing gum was previously determined with ICP-MS.

b Dilution is only indicative and needs to be adjusted to obtain 1000-2000 spikes per scan time.

After sonication, it was verified that the pH of the samples was above 6 to avoid any agglomeration which occurs close to the isoelectric point of titanium dioxide in water (Kosmulski, 2002; Suttiponparnit et al., 2010; Verleysen et al., 2020). Sonicated suspensions were then diluted with ultrapure water and analysed with CLS, spICP-MS and TEM, as detailed in section 2.4. The dilution factors are included in Table 3, which provides an overview of all sample preparation steps.

### 2.4 Instruments and instrumental settings

#### 2.4.1 Single-particle ICP-MS analysis

A PerkinElmer Nexion 300D quadrupole ICP-MS, equipped with a SC fast peristaltic pump, a Meinhard concentric nebuliser, a glass cyclonic spray chamber and a standard quartz torch (2.5 mm i.d.) operating in standard mode was used for spICP-MS analysis (PerkinElmer, Waltham, MA, US). The operating conditions were optimised every day to achieve maximum sensitivity. For the settings of all parameters and data acquisition, the Nano Application Module (Version 1.1) of the Syngistix™ software was used. The dwell time was set at 100 μs, and the total data acquisition time at 60 s. The transport efficiency (TE) can be determined either based on measured particle frequency or based on measured particle size. Due to the unavailability of a reference material certified for number-concentration, in this work, the TE was determined following the ‘particle size’ approach (Pace et al., 2011). The exact flow rate of the peristaltic pump required for the determination of the transport efficiency was measured daily and was approximately 0.17 mL min^−1^. A 63 nm gold NP suspension with a concentration of approximately 100,000 particles mL^−1^ and solutions of dissolved gold (blank and four solutions ranging from 1 to 10 μg L^−1^) were prepared by diluting the stock solutions with ultrapure water. The dissolved ionic gold standard for ICP-MS (1 g L^−1^ in 5% HCl) and gold nanospheres with a nominal diameter of 63 ± 7 nm (43.45 μg mL^−1^ in aqueous 2 mM sodium citrate, zeta-potential −55 mV) were purchased from Sigma-Aldrich (St. Louis, MO, US) and NanoComposix (NanoComposix, NanoXact™, Product Number AUCN60, San Diego, CA, US), respectively. The transport efficiency ranged from 11.3% to 13.8%. For the determination of titanium dioxide nanoparticles, the titanium-48 isotope was monitored setting the mass fraction to 60% and density to 3.9 g cm^−3^ (density of anatase). Anatase is the most frequently used crystalline form for food-grade titanium dioxide (Younes et al., 2019). Possible polyatomic and isobaric interferences such as ^32^S^16^O and ^48^Ca (0.187% abundance) appear as continuous background from which the discontinuous signals generated by titanium dioxide particles can be distinguished (Bucher & Auger, 2019; Peters et al., 2014). In this study, the sugar coating of the studied food samples had negligible levels of potentially interfering elements and the use of ion-molecule chemistry or other methods of interference removal was therefore not necessary. A 5-point calibration curve ranging from 0 to 50 μg L^−1^ dissolved titanium in 0.1% HNO_3_ was used for the size calibration. The dissolved ionic titanium standard for ICP-MS (1 g L^−1^ in 2% HNO_3_) was purchased from Sigma-Aldrich (St. Louis, MO, US). The diluted (1:60,000) sample suspensions resulted in approximately 1000–2000 particles detected per 60 s scan time which can be considered as being the minimum amount of particles which should be analysed to produce statistically robust particle size distributions. The smallest nanoparticle that can be detected using spICP-MS is determined by the sensitivity of the ICP-MS system and the ability to differentiate particle signals from the background signal. It corresponds to the point where the extrapolated particles signal intensity equals the background plus 3 times the standard deviation (Laborda, Bolea, & Jiménez-Lamana, 2014). In our case the detection limit for size was around 35 nm, with small variations depending on the instrument's daily performance.

#### 2.4.2 Transmission electron microscopy (TEM) analysis

Dispersions required cleaning to remove colorants and sugar before application to TEM grids. Suspension-aliquots of 500 μL of both candy and chewing gum extracts were transferred into 1.5 mL Eppendorf® vials and centrifuged at 6000 rpm (at approximately 2000 g) for 2 h. The supernatant was removed, and the pellet resuspended in 500 μL of ultrapure water. A second centrifugation step of 2 h at 6000 rpm (2000 g) was applied. After removal of the supernatant, the pellet was resuspended in 17 μL of ultrapure water. Approximately 15 μL of this dispersion were finally applied onto pioloform- and carbon-coated 400 mesh copper grids (Agar Scientific, Essex, UK), which were pre-treated with Alcian blue. The grids were then left in contact with the suspension for 10 min. Hereafter, the grids were blotted to remove excess sample and air dried at room temperature.

The samples were imaged in bright-field TEM mode using a well-aligned Tecnai G2 Spirit electron microscope (Thermo Fisher Scientific, Eindhoven, the Netherlands) with the Biotwin lens configuration operating at 120 kV. In general, the methodologies described by De Temmerman et al. (De Temmerman et al., 2012) and Verleysen et al. (Verleysen et al., 2020) were followed. To assure the precision and accuracy of the TEM measurements and to relate them to the international system of units (SI), the calibration of the TEM is critical. This was realised with the support of the guidance document ISO 29301 (International Organization for Standardization, 2017b) for magnification calibration of the images over the applied magnification range. Calibration was done at two levels. Firstly, the reference materials applied for magnification calibration possess a periodic structure that makes it suitable for automated calibration using specialized calibration software (Thermo Fisher Scientific), according to the manufacturer's instructions. In addition, a complementary evaluation based on the measurement of the size of a certified reference material with an SI-traceable size was performed periodically (Verleysen et al., 2019).

Images were recorded using the TEM imaging & analysis (TIA) software (Thermo Fisher Scientific). These SER- and EMI-formatted micrographs were converted to TIF format using the TIA software. The magnification of the micrographs and the number of particles (micrographs) were determined such that the images are suitable for subsequent descriptive and quantitative image analyses. The pixel size and the associated magnification was determined based on the criterion of Merkus (Merkus, 1975) as applied to the titanium dioxide representative test materials NM-100 by Verleysen et al. (Verleysen et al., 2019). The upper size detection limit was limited to one tenth of the image size in accordance with ISO 13322-1 (International Organization for Standardization, 2014). The number of particles required to estimate the key quantitative parameter(s) within a given confidence level was determined based on the method proposed by Verleysen et al. (Verleysen et al., 2019). This method is based on the relation between the number of measured particles and the measurement uncertainty. To assure unbiased random image collection for quantitative TEM analysis, the systematic micrograph selection procedure described by De Temmerman et al. (De Temmerman et al., 2012) was applied. Subjectivity in the selection of particles by the microscopist was avoided by taking the micrographs randomly and systematically, at positions pre-defined by the microscope stage and evenly distributed over the entire grid area. When the field of view was obscured, e.g., by a grid bar or an artefact, the stage was moved sideways to the nearest suitable field of view. The images were then analysed as described by Verleysen et al., 2019 (Verleysen et al., 2019). The ‘ellipse fitting mode’ of the NanoDefine ParticleSizer plugin of Image J software (NanoDefine, 2016) was applied to measure constituent particles. This fits an ellipse to the selection and determines the primary (major) and secondary (minor) axis of the best fitting ellipse. For the examined material, the primary (major) axis is a good estimate for the (maximum) Feret diameter (Fmax) (defined as the longest distance between any two points along the selection boundary, which is also known as the maximum caliper diameter), and the secondary (minor) axis is a good estimate of the minimum Feret diameter (Fmin) (defined as the minimum caliper diameter). The quantitative image analysis was performed for the measurands Fmin, Fmax and the aspect ratio (AR). For each specimen, at least 500 particles were analysed.

#### 2.4.3 Centrifugal liquid sedimentation (CLS)

The CLS analysis was used only for the assessment of the minimum sonication conditions required for sample preparation (see section 2.3). CLS measurements were performed using a line start CPS disc centrifuge (CPS Instruments Europe, Oosterhout, The Netherlands) equipped with a 405 nm laser, using an 8–24% sucrose gradient at a rotation speed of 18,000 rpm. Polyvinyl chloride (PVC) particles with a diameter of 237 nm were used for calibration before each single measurement. Of the diluted suspensions (see Table 3), aliquots of 100 μL were injected into the disk of the centrifuge. An absorption of 0.075 and the refractive index and density values of anatase (2.49 and 3.9 g cm^−3^, respectively) were used as the input parameters for the calculations. The choice of these values was however of secondary importance, as the impact of the sonication conditions on the agglomeration/aggregation state of the extracted particles was qualitatively assessed by comparing the particle size distributions (PSDs).

### 2.5 Restricted interlaboratory comparison study

The objective of this restricted interlaboratory comparison study was to obtain information on the proposed method's applicability in and transferability to a variety of laboratories as well as on its analytical performance.

#### 2.5.1 Participants and distribution of samples

Seven laboratories, listed in Table 4, participated in the restricted ILC. The laboratories were selected in the frame of a broader activity coordinated by the Joint Research Centre of the European Commission, the objective of which was to assist Member State Control Laboratories in the implementation of current legislation concerning (engineered) nanoparticles in food matrices. The seven laboratories were identified as those food control and food research laboratories with specific experience in the analysis of titanium dioxide with spICP-MS. One of the laboratories participated in the ring-trial with two different instruments.

**Table 4:** Participants at the restricted interlaboratory comparison study, instruments, instrumental settings and software packages used by each laboratory.

Name of Laboratory	Country	Instrument	Software	Pump Flow Rates [mL min^−1^]	Dwell Time [μs]	Transport Efficiency [%]
Sciensano	Belgium	Agilent 8800	Rikilt Calculation Sheet	0.473	3000	6.5
Max Rubner-Institut (MRI) – Karlsruhe	Germany	Thermo iCAP Q	Thermo Qtegra with npQuant plugin &Rikilt Calculation Sheet	0.31	3000	7.3
National Food Institute, Technical University of Denmark	Denmark	Agilent 8900	Single Nanoparticle Application Module of the Agilent ICP-MS MassHunter software 4.5	0.339	100	5.2
Service Commun des Laboratoires (SCL)	France	Perkin Elmer Nexion 300 and Perkin Elmer Nexion 2000	Syngistix V1.1 and Syngistix V2.3	0.18	100	9–12
Istituto Superiore di Sanità (ISS) - Rome	Italy	Perkin Elmer Nexion 350D	Syngistix V2.3	0.277	100	11.2
WFSR - Wageningen University & Research	Netherlands	Perkin Elmer Nexion 350D	Syngistix V1.1	0.171	100	11.2
Joint Research Centre of the European Commission - Ispra/Italy	European Commission	Perkin Elmer Nexion 300D	Syngistix V1.1	0.169	100	12.0

#### 2.5.2 Organisation aspects of the restricted interlaboratory comparison study

The interlaboratory comparison study was executed in 2019. Each participant received 3 Falcon™ tubes with 6 button-shaped candies each, 3 tubes with 3 chewing gum dragées each and 1 g of pristine titanium dioxide powder. While the analysis of the food samples was requested for all participants, the analysis of the pristine material was on a voluntary basis. In addition to the sample materials, aliquots of a well-characterised 63 nm gold nanoparticle suspension (NanoXact Gold NanoSpheres Bare, 63 nm, 43.45 μg mL^−1^ in aqueous 2 mM sodium citrate, Product Code AUCN60, NanoComposix Europe, Prague, Czech Republic) were distributed for the determination of the transport efficiency. These materials were distributed to the participants together with a standard operating procedure and a standardised template for results.

#### 2.5.3 Standard operating procedure (SOP) used for the interlaboratory comparison study

The sample preparation procedure was identical to that detailed in section 2.3. The food sample suspensions were bath sonicated (at the maximum power for 600 s), whereas the suspended pristine titanium dioxide powder required probe-sonication (delivered energy: 10 kJ; delivered acoustic power: 18 Watt) to achieve de-agglomeration of the majority of particles. The final dilution step before the spICP-MS analysis varied depending on the instrument used by each of the participants. The instrumental settings were to a large extent those detailed in section 2.4.1. Depending on the type of instrument, the dwell time and the scan-time in some cases required adjustment for the spICP-MS analysis. The standard operating procedure can be found in the supplementary material (SM4).

In parallel to the spICP-MS analysis performed by all participants, a set of samples was additionally analysed by transmission electron microscopy in one laboratory only. The descriptive TEM analysis, based on representative and selected electron micrographs, was applied to verify the specimen preparation, to demonstrate the presence of (nano)particles, and to describe the nanoparticle shape and aggregation/agglomeration status as well as the presence of impurities, as assessed by visual observation of aberrant structures. The quantitative TEM analysis provided number-based distributions of characteristic parameters, including the minimum external dimension of the constituent particles, estimated as, e.g., the minimum Feret diameter (Haider et al., 1998), in line with the EC definition recommendation of nanomaterials. Two image analyses were performed on each TEM specimen: one measuring the properties of agglomeration and one measuring the constituent particle properties. Expanded uncertainties (95%) on the measurements were provided as determined in top-down validation studies estimating the repeatability and intermediate precision, including calibration and trueness uncertainties (Verleysen et al., 2019).

#### 2.5.4 Statistical evaluation of the results

All results were statistically evaluated using a validated commercial software package (ProLab, Quodata, Dresden, Germany). The consensus value, repeatability and reproducibility of the method were calculated following robust statistics according to the indications laid down in ISO 5725, Part 5 (International Organization for Standardization, 1998). The robust method proposed in this section of ISO 5725 considers all the data and assigns different weightings to level the impact of outliers on the overall result. For the purpose of comparison, the results were also calculated using Part 2 of the standard ISO 5725 (International Organization for Standardization, 2004). In ISO 5725-2, outliers and strugglers are excluded from the statistical evaluation. A number of measurands were considered for the evaluation of the interlaboratory comparison study. For spICP-MS, these included the particle mean diameter, the most frequent diameter (mode), the percentage of particles (in number) with a diameter below 100 nm, the smallest particle diameter (lower bound, D0), the largest particle diameter (upper bound, D100) and various cumulative particle size distribution parameters (by number) such as D10, D50, D99.5 and D99.8. Although the focus of this interlaboratory comparison study was determining the particle size distribution, the particles’ number concentration was also examined and statistically evaluated as an additional assessment.

## 3 Results and discussion

### 3.1 Sample preparation – role of sonication conditions on the particle size distribution

The role of sonication intensity on the particle agglomeration/aggregation state was assessed with centrifugal liquid sedimentation and single-particle ICP-MS.

#### 3.1.1 Centrifugal liquid sedimentation

**Fig. 1:**
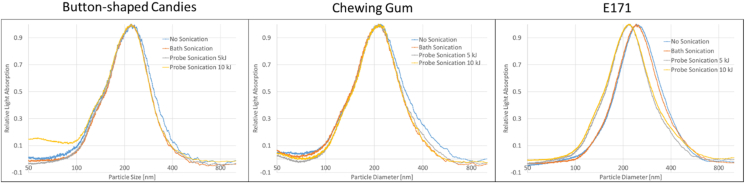
Particles size distributions determined by centrifugal liquid sedimentation for all three sample materials. Each sub-figure includes the size distributions for the four sonication conditions.

Fig. 1 shows the particle size distributions (PSDs) for all three sample materials. Each of the sub-images include the overlaid size distributions of the non-sonicated, the bath-sonicated and the probe-sonicated (5 and 10 kJ) particle suspensions.

While the particle size distributions of the particles extracted from the button-shaped candies and from the chewing gum match well for all four sonication conditions, a difference can be observed for suspended pristine titanium dioxide. The particle size distributions of the non-sonicated and the bath-sonicated suspensions show a relevant shift towards larger particles. Comparing the peak-apices of the non-sonicated/bath sonicated with that the probe-sonicated distribution, the difference corresponds to approximately 35 nm. In contrast, the probe-sonicated suspensions follow a particle size distribution profile of generally smaller particles, comparable to those obtained for the candies and the chewing gum. The particles are submitted to attractive and repulsive forces. Agglomeration occurs when the attractive forces are predominant, and the smaller the particle size is, the higher these forces (Henry et al., 2013). The forces holding agglomerates together are weak forces, for example van der Waals forces, as well as simple physical entanglement (He, Wan, & Tokunaga, 2008; Kobayashi, Juillerat, Galletto, Bowen, & Borkovec, 2005). While the particles in aggregates are strongly bound and aggregation is often considered as non-reversible, agglomerates are brittle structures, which can be broken down and rebuilt depending on the strength of the external forces. In the pristine titanium dioxide material, the particles are closer together than in the candies and the chewing gum, resulting in stronger attractive forces among the particles and a higher agglomeration propensity. Differences in particle concentration, steric stabilisation by food components and pH effects can also explain the need for a stronger sonication to obtain a stable dispersion of the pristine material.

#### 3.1.2 Single-particle ICP-MS

**Fig. 2:**
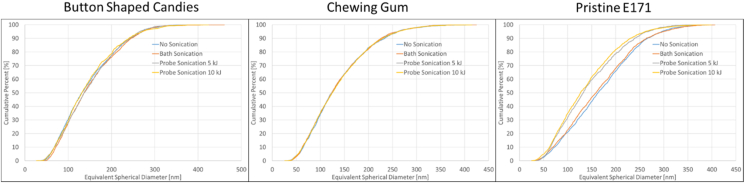
Single-particle ICP-MS – Cumulative number size distributions of titanium dioxide particles under four sonication conditions in button-shaped candies, chewing gum and pristine E 171

The second analytical technique used to assess the impact of the different sonication conditions on the agglomeration state of the titanium dioxide particles in the three tested matrices was single-particle ICP-MS. Fig. 2 represents the cumulative particle size number distributions of the four sonication conditions. The cumulative curves of pristine E 171 clearly show a distribution difference between probe-sonicated samples and bath-sonicated samples. The probe-sonicated samples result in a cumulative number distribution that is shifted towards smaller particle sizes.

The results confirm the findings obtained with centrifugal liquid sedimentation. In conclusion, for relatively simple matrices - such as the candy and chewing gum used in this study, where the titanium dioxide is present only in the outer sugar shell - bath sonication is sufficient to reach the highest, achievable degree of dispersed constituent particles with this protocol. For pristine E 171, on the other hand, probe sonication is required.

### 3.2 Results of the restricted ring trial

Of the seven laboratories which participated in the interlaboratory comparison study, one analysed the three samples with two different instruments; since independent data-sets were required, the statistical data evaluation was performed with only one of them (data generated with the PerkinElmer Nexion 2000 was excluded). Therefore, in total 7 data-sets were used for the statistical evaluation.

#### 3.2.1 Overview of the results

The results were evaluated according to ISO 5725-5 without the exclusion of outliers. Table 5 includes the performance parameters of the interlaboratory comparison study (spICP-MS data).

**Table 5:** Results of the collaborative trial according to ISO 5725, part 5.

Measurand	Test material	X_pt_	U (X_pt_)	R	r	RSD_R_ [%]	RSD_r_ [%]
Mean Particle Diameter [nm]	Button shaped candies	158	9.1	12.3	3.1	7.8	2.0
	Chewing gum	149	10.3	14.4	5.8	9.7	3.9
	E 171	163	15.5	19.2	3.6	11.8	2.2
Most Frequent Particle Diameter (Mode)[nm]^b^	Button shaped candies	104	3.5	7.1	6.5	6.8	6.3
	Chewing gum	97	5.2	10.0	8.8	10.3	9.1
	E 171	95	8.6	11.4	5.2	12.0	5.5
Particles < 100 nm [%]	Button shaped candies	26.0	3.4	4.6	1.0	17.6	3.7
	Chewing gum	30.2	4.0	5.6	2.4	18.7	7.8
	E 171	27.7	3.9	5.0	2.0	18.1	7.2
D10 [nm]	Button shaped candies	71	4.0	5.7	2.5	7.9	3.5
	Chewing gum	66	4.7	6.8	3.3	10.2	5.1
	E 171	69	2.9	4.7	3.8	6.9	5.5
D50 (Median)[nm]	Button shaped candies	146	7.8	10.7	3.3	7.3	2.3
	Chewing gum	136	8.7	12.6	6.3	9.3	4.6
	E 171	149	10.1	12.7	3.7	8.6	2.5
D99.5 [nm]	Button shaped candies	415	52.5	71.1	18.5	17.1	4.5
	Chewing gum	405	43.2	59.3	18.9	14.6	4.7
	E 171	428	81.5	100.6	15.6	23.5	3.6
D99.8 [nm]	Button shaped candies	455	72.7	97.5	19.7	21.4	4.3
	Chewing gum	457	78.4	106.5	29.3	23.3	6.4
	E 171	475	103.6	127.8	18.4	26.9	3.9
Smallest Particle Diameter, Lower bound (D0)[nm]	Button shaped candies	38	13.4	17.8	1.7	46.5	4.6
	Chewing gum	38	13.6	18.0	1.6	47.0	4.2
	E 171	38	16.1	19.8	1.9	52.5	4.9
Largest Particle Diameter, Upper bound (D100)[nm]	Button shaped candies	482	150.5	202.9	48.0	42.1	10.0
	Chewing gum	461	134.4	183.0	53.6	39.7	11.6
	E 171	561	161.6	206.8	73.5	36.9	13.1
Total number of particles in sample^a^	Button shaped candies	4.1E11	9.7E10	1.3E11	3.8E10	32.2	9.2
	Chewing gum	3.5E11	9.1E10	1.3E11	4.6E10	35.9	13.1
	E 171	1.1E12	3.8E11	4.7E11	1.3E11	43.1	11.4
Particle Number Conc. [# g^−1^]	Button shaped candies	7.4E10	1.7E10	2.3E10	7.7E09	31.4	10.3
	Chewing gum	8.3E10	2.2E10	3.0E10	1.1E10	36.5	12.7
	E 171	2.7E13	9.3E12	1.2E13	3.3E12	43.5	12.4

a Sample intended as the total amount of candy, total amount of chewing gum and the amount of weighed pristine material.

b In the presence of multimodal distributions, the value with the highest occurrence frequency was used for the evaluation of the results - X _pt_: robust average or consensus value; U (Xpt): uncertainty in the consensus value, calculated as 2*standard error; R: reproducibility; r: repeatability; RSD_R_: relative standard deviation in the reproducibility; and RSD_r_: relative standard deviation in the repeatability.

In the absence of appropriate data from other ILC studies, the precision of the method was calculated using the robust reproducibility standard deviation (R) for all measurands. The assessed method performance characteristics are the within-laboratory precision, expressed as the relative repeatability standard deviation in (RSD_r_), and the between-laboratory precision, expressed as the relative reproducibility standard deviation in (RSD_R_). Repeatability and reproducibility were below 10% and 25% respectively for most measurands except for the smallest (D0) and largest (D100) particle sizes and the particle number concentration.

The larger distribution obtained for the measurand ‘particle number concentration’ may be explained by slight differences in the sample preparation method between the individual samples (e.g., dissolution of the titanium dioxide containing sugar shell, dilution steps, and particle adsorption onto sidewalls of tubes or tips) and the possible loss of particles during analysis (adsorption onto tubing or other parts of the instrumentation). These small deviations do not remarkably affect the particle's sizing; they can however strongly influence the particles' number concentration. Waegeneers and co-authors conducted a study in which they validated in-house a method for the sizing and quantification of silver nanoparticles in confectionery samples. In this study, the authors examined the uncertainties associated with sample preparation that could have an impact on the repeatability variation of the results. The heterogeneous content of silver among candies and a non-optimised sample dispersion protocol were identified as the main sources. Moreover, the authors of that study concluded that the non-optimised dispersion protocol did not have an impact on the particles' size distribution, but on the particle concentration (Waegeneers, De Vos, Verleysen, Ruttens, & Mast, 2019). Other possible reasons for a broader distribution of results are discussed in the following paragraphs.

Although not directly comparable due to the different types of titania used, the particle size distributions of pristine and extracted E 171 found in this interlaboratory comparison study are generally in good agreement with those published in (non ILC) studies conducted in the past. In most of these studies only a few measurands were reported. Representative values for the percentage of particles (in number) with a diameter below 100 nm in pristine E 171 can be extracted from the data submitted by the industry to the European Food Safety Authority (EFSA) in 2016 (Younes et al., 2019). These values determined by electron microscopy ranged from 11 to 39%. In two studies E 171 was characterised with spICP-MS. Helsper and co-authors (Helsper et al., 2016) analysed 7 food grade pigments and found particles' size to range approximately between 50 and 500 nm and the mode ranged from 150 to 220 nm. Verleysen et al. (Verleysen et al., 2020) analysed 12 pristine E 171 materials and found median equivalent size diameters ranging from 83 to 125 nm. Only a few research groups determined the size distribution of titanium dioxide particles in extracts of confectionery by spICP-MS. Bucher et al. (Bucher & Auger, 2019) measured the particle size distribution of food grade titanium dioxide in coconut syrup containing E 171 as a white food colouring, in hard wedding candies made of almonds covered with sugar and E 171 and in soft jelly candies. The most frequent size was found to be around 100 nm. The particles' size ranged from 30 to 400 nm. Vidmar and co-workers (Vidmar et al., 2019) analysed E 171 in cake decoration. The size distribution was between 30 and 450 nm, with a median diameter of 54 nm and 70% of the particles (in number) < 100 nm. Candas-Zapico and co-workers (Candás-Zapico et al., 2018) analysed the particle size distribution of food grade titanium dioxide in chewing gum samples using a triple quadrupole ICP-MS operated in various measuring modes (cell- and reaction-gases) and following a similar sample preparation protocol as the one described in this work. They found particle size to range between 30 and 200 nm. Approximately 40% of particles were found to be smaller than 100 nm. Geiss et al. (Geiss et al., 2019) characterised the size distribution of titanium dioxide particles contained in eight confectionery products. They found the particles’ size to range between 40 and 350 nm. The relative frequency of particles below 100 nm was found to be 12–19%. Peters et al. (Peters et al., 2014) analysed the particle size distribution of E 171 in chewing gum. Particle diameters below 100 nm ranged from 5 to 10%. The size range was approximately 50–600 nm.

##### 3.2.1.1 The impact of using different instruments and software-packages

Four out of the seven ILC study participants used instruments from the same manufacturer which were similar models, within the same brand, using similar software versions (see Table 4). The use of non-identical instrumentation can possibly introduce a higher level of variation in the results. Within the same brand and model for instance, the timing parameters (dwell time and scan time) are homogenously set. In addition, the use of data processing software from the same manufacturer and applying the same data treatment algorithm, can also contribute to a narrower distribution of the results. For example, not all software packages provided the results for all measurands directly on the output screen and therefore required data exportation and external data processing with tools such as the ‘Single Particle Calculation tool’ (https://www.wur.nl/en/show/Single-Particle-Calculation-tool.htm) developed by the Wageningen Food Safety Research Institute in the Netherlands or other commercial data-treatment packages. Most instruments in this ILC study allowed for setting the dwell time at 100 μs? For two of the other instruments, the lowest dwell time that could be used was 3000 μs? This difference had a direct impact on the sample preparation procedure. Longer dwell times required a higher dilution and longer scan-times to obtain the same number of spikes (1000–2000 spikes/scan-time). In the absence of an increased background due to dissolved species (as in these samples), the need to set longer dwell times should however not strongly affect the overall sizing and counting results (Abad-Álvaro et al., 2016). These, and other, differences between instruments made it difficult to prepare a detailed standard operating procedure valid for all instruments. Some of the instructions required brand- and model-specific adaptation by the operators. Another difference that can be observed in Table 4, is that the transport efficiencies of those instruments used by most of the participants is on average almost twice that of the other instruments. This considerable variation can be explained by the different instrumental set-ups (sample introduction systems). The correct setting of the transport efficiency is fundamental for not only the determination of particle number-concentration but also for the correct sizing of the particles. An error in the determination of the transport efficiency has, however, a stronger impact on the particle number than on particle sizing, which might explain the broader distribution as well. The verification of a correctly set transport efficiency is of fundamental importance. This can however only be made against a certified reference material that is certified for both particle size and particle number-concentration. Such a material is however currently not available.

##### 3.2.1.2 Most frequent particle diameter (mode) and smallest/largest detected particle diameter (particle size range)

Particle size range.

The lower and upper bounds of the particle size distribution are two of the measurands considered in this interlaboratory comparison study for which the distribution in the results is broader than average, when compared to the other measurands. For titanium dioxide E 171, the lower bound of the size-distribution coincides with the size detection limit which for spICP-MS depends on the nature of the analysed element (Aznar et al., 2017). It is defined as the nanoparticle size that can be distinguished from the continuous background and depends on, among others, the transport efficiency, the dwell time used, the concentration of the dissolved analyte, the spectroscopic interferences, the way the software subtracts the signal of the dissolved element from the particle signal, the type of mass spectrometer (e.g., single vs. triple quadrupole) and the daily instruments' performance. Some of these parameters can vary between instruments, between samples (matrix) and day-by-day on the same instrument (e.g., small differences in the instrument's daily performance). These factors might explain some of the differences observed in this study. The results for the largest particle diameter (upper bound) are even more broadly distributed. The main reason can be identified as the non-harmonised application of the upper counting limit. If no such limits are defined, single isolated very large particles can strongly influence the reported values. To exclude such single isolated large - presumably agglomerated/aggregated - particles, defined D-values of the cumulative particle (number) distribution curve can be reported instead of the effectively largest particle diameter directly provided by the instrument's software.

Based on the data of one of the participating laboratories, Table 6 shows the impact of the selection of D-values on the resulting sizes in the particle size distribution curve for the three selected sample materials.

**Table 6:** Impact of cumulative D-values on resulting largest particle diameter (the example using single data-sets from one laboratory only).

D-Values	Absolute number of particles that would be excluded by setting this D-value^a^	Button-shaped Candies [nm]	Chewing Gum [nm]	Pristine E 171 [nm]
D90	200	251	222	260
D95	100	284	257	294
D99	20	338	330	340
D99.5	10	363	350	363
D99.8	4	375	371	399
D100	0	453	401	509

a Assuming total particle number of 2000 per scan-time.

**Fig. 3:**
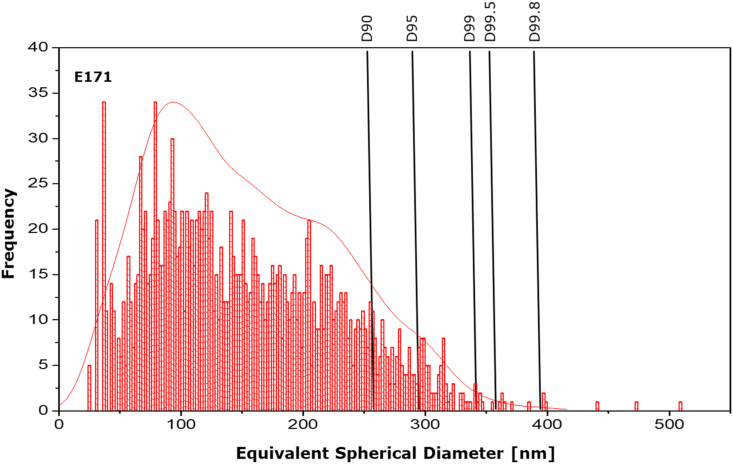
Particle number-size distribution of pristine titanium dioxide particles measured by spICP-MS and the impact of choosing various D-values on the derived largest particle diameter (upper bound).

Especially for the pristine E 171 material, for which the likelihood that constituent particles agglomerate is higher, the derived largest particle diameter decreases by approximately 100 nm when choosing a D-value of 99.8. Fig. 3 shows that 3 out of 2000 particles are responsible for a 100 nm size range extension.

Looking at these three cases, it appears that reporting D99.5 or D99.8 instead of the effectively largest particle diameter (D100) would result in a less broad distribution in this measurand among the seven reporting laboratories. In addition to the upper bound provided by the instrument's software considering all detected particles (called ‘largest particle diameter (upper bound), D100’ in the table), Table 5 includes the D99.5 and D99.8 values obtained for all participating laboratories. The standard deviation of the reproducibility for both D99.5 and D99.8, are approximately halved compared to the reproducibility value when the upper bound considers all particles detected during the scan-time. This result confirms that using D99.5 or D99.8 instead of the value provided by the instrument, considerably narrows the variability in the reported largest particle diameter values.

Most frequent particle diameter (Mode).

The ‘most frequent particle diameter’ values originally reported by the participants were either approximately 100 nm or distinctly smaller (approximately 60–70 nm). The most frequent particle diameter according to TEM analysis reveals a value of approximately 100 nm. One of the reasons for the discrepancies in the reported values was the way the absolute frequencies were displayed (histograms). Some of the single-particle software applications installed on ICP-MS instruments did not display the number-size distributions at constant bin-sizes. The appearance of the number-size distributions is however driven by the choice of the bin-size. To overcome this dependency, kernel density estimates were determined for all the particle size-distributions in this interlaboratory comparison study. Since they are bin-size independent, they therefore allow the reporting of the most frequent particle size in a standardised way. The weakness of this approach is that kernel density estimates can only be determined from the size-distribution exported from the instrument software into specific data processing software tools (e.g., Origin or Python). Instructions on how to generate Kernel density plots in Origin are included in the standard operating procedure (SM4).

### 3.3 Confirmatory analysis with transmission electron microscopy

The TEM method used in this study was previously validated by Verleysen et al. (Verleysen et al., 2019). Measurement uncertainties were assessed against NM-100, a representative test material very similar to E 171 (Rasmsussen et al., 2014) and the trueness of the method was determined against the European reference materials ERM-FD100 (colloidal silica, 20 nm) and ERM-FD304 (colloidal silica, 40 nm).

#### 3.3.1 Descriptive analysis

**Fig. 4:**
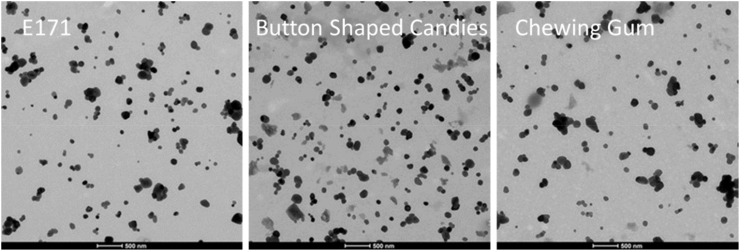
Representative TEM images of all three sample materials.

Both constituent particles and agglomerates/aggregates were found in all specimens of pristine E 171, chewing gum and button-shaped candies. The shape of the pristine particles was ellipsoidal. While the pristine titanium dioxide was relatively pure, the other two sample extracts contained background impurities, which were not entirely eliminated during the sample preparation. These can be observed as light-grey areas in Fig. 4.

The blank samples did not show any particles. In the case of the pristine E 171 material (the only probe-sonicated material), this observation indicates that either the sonication tip did not release any particles during sonication or the number of particles released from the tip was below the detection limit.

#### 3.3.2 Quantitative analysis

**Fig. 5:**
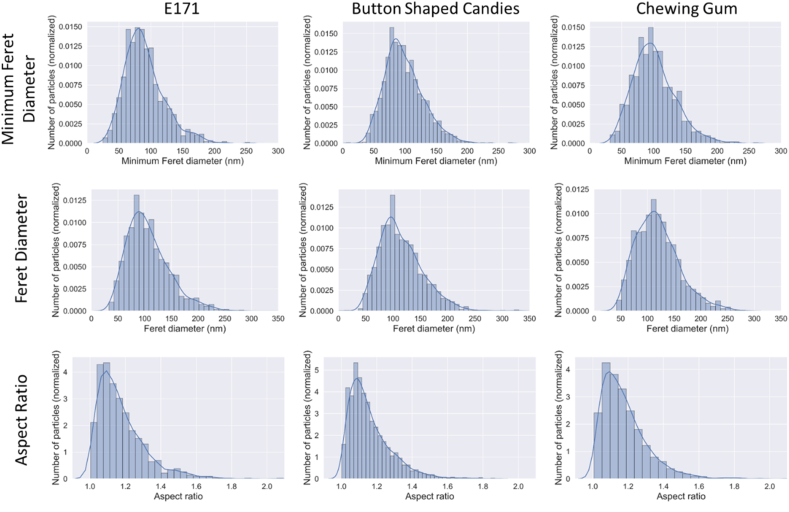
Normalised number-based distributions (histograms) and Kernel density estimations (solid line) of the minimum Feret diameter, the (maximum) Feret diameter and the aspect ratio of the constituent particles detected in all three sample matrices.

A magnification suitable for the quantitative TEM analysis could be determined by applying the criterion of Merkus, such that the large majority of the analysed particles were larger than the lower limit of quantification (LLOQ = 11.5 nm) and smaller than the upper limit of quantification presented (ULOQ = 478 nm). Particles could be distinguished from the background based on mass-thickness contrast. A large majority of particles were identified correctly and could be measured using ellipse fitting (The images are available in the supplementary material (SM5)). Histograms showing the constituent particle size distribution of Fmin, Fmax and the aspect ratio for each sample are shown in Fig. 5.

The statistics for each TEM sample measurement are presented in Table 7. The mean particle diameter, the median particle diameter and the most frequent particle diameter were determined with both techniques (spICP-MS and TEM). Table 8 includes these measurands determined by both techniques. In spICP-MS, the size was determined by converting the mass into diameter, assuming perfect particle sphericity. For the comparison of the values obtained by the two techniques, the minimum Feret diameter (TEM) is compared to the equivalent spherical diameter (ESD) determined with spICP-MS. The reason for this choice is that for near spherical particles the ESD can be considered as a proxy of the minimal external diameter, as is the minimum Feret diameter.

**Table 7:** Summary of the mean, median and mode values of the minimum Feret diameter, the Feret diameter and the aspect ratio and their related uncertainties (95% confidence interval) obtained from the quantitative TEM analysis of the constituent particles of all three sample matrices.

Test Materials	Minimum Feret Diameter [nm]			Feret Diameter [nm]			Aspect Ratio			Number of analysed particles
	Median	Mean	Mode	Median	Mean	Mode	Median	Mean	Mode	
Pristine E 171	85 ± 7	90 ± 8	81 ± 7	100 ± 9	107 ± 10	89 ± 8	1.14 ± 0.04	1.18 ± 0.04	1.10 ± 0.04	1158
Button-shaped candies	94 ± 8	99 ± 8	85 ± 8	108 ± 10	115 ± 10	96 ± 9	1.13 ± 0.04	1.16 ± 0.04	1.09 ± 0.04	1793
Chewing gum	98 ± 8	101 ± 9	95 ± 8	115 ± 10	119 ± 11	112 ± 10	1.15 ± 0.04	1.18 ± 0.04	1.10 ± 0.04	1347

**Table 8:** Comparison of the average mean particle diameter, most frequent particle diameter (mode) and median particle size values determined by TEM and spICP-MS (diameter ± uncertainty^c^).

	Mean Particle Diameter [nm]		Most Frequent Particle Diameter [nm]		Median Particle Diameter [nm]	
	spICP-MS^b^	TEM^a^	spICP-MS^b^	TEM^a^	spICP-MS^b^	TEM^a^
Pristine E 171	163 ± 15.5	90 ± 9.9	95 ± 8.6	81 ± 8.9	149 ± 10.1	85 ± 9.4
Button-shaped candies	158 ± 9.4	99 ± 10.9	104 ± 3.5	85 ± 9.4	146 ± 7.8	94 ± 10.3
Chewing gum	149 ± 10.3	101 ± 11.1	97 ± 5.2	95 ± 10.5	136 ± 8.7	98 ± 10.8

a Minimum Feret diameter. TEM analysis done only in one laboratory.

b Expressed as the equivalent spherical diameter. The mean of all values (7 laboratories) considered in the interlaboratory study (statistical data treatment according to ISO 5725 Part 2).

c TEM: expanded measurement uncertainty; spICP-MS: uncertainty in the consensus value.

**Fig. 6:**
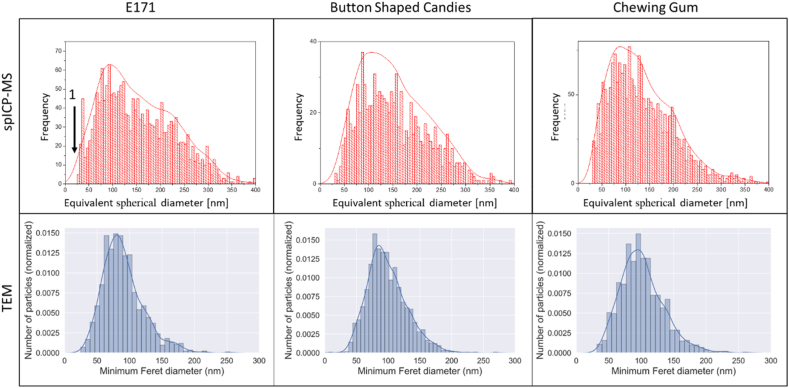
Number-based particle size distributions determined by TEM and spICP-MS. The continuous line represents the Kernel-density estimate.

The results show that the mean and the median particle diameter determined by spICP-MS is in all cases higher than the sizes determined by TEM, whereas for the most frequent particle size (mode), the values are closer together, and no clear trend can be observed. The discrepancies between the two techniques concerning the mean particle diameter, and to some extent also the median, can be explained by the size detection limit for spICP-MS and by the fact that a part of the analysed particles remained agglomerated/aggregated even after sonication. As stated above spICP-MS cannot distinguish between constituent and agglomerated/aggregated particles hence an agglomerate of constituent particles is detected as one larger particle. In the presence of agglomerates, the number of larger particles is therefore overestimated, while the number of smaller particles might be underestimated when using spICP-MS in comparison to TEM. Fig. 6 includes the particle size distributions determined with both TEM and spICP-MS. While the upper bound of particles detected by TEM ends at approximately 250–300 nm; with spICP-MS, particles can be observed up to a size of approximately 350–400 nm.

Moreover, the size-threshold, below which no particles can be measured with spICP-MS, is dictated by the size-detection limit, which strongly depends on the nature of the investigated element (Aznar et al., 2017; Lee et al., 2014). For titanium dioxide, the size detection limit lies between 30 and 35 nm, depending on the type of instrument. The TEM analysis however shows that E 171 does contain a small fraction of particles below this size. The area in which particles are not counted are marked with the number one in Fig. 6. Consequently, the number of smaller particles is slightly underestimated, and the mean/median sizes are overestimated with spICP-MS. Furthermore, spICP-MS does not allow to directly determine the minimum Feret diameter frequently requested in the regulatory context (Verleysen et al., 2020). Finally, yet importantly, consideration needs to be given to the fact that the values reported for spICP-MS are the average values of all seven laboratories, while the TEM values were generated in one laboratory only. This might add uncertainty to the comparison of values obtained by the two analytical techniques.

Table 8 shows that, for the pristine titanium dioxide, the TEM analysis would identify the material as being a nanomaterial (median diameter < 100 nm) according to the EC recommended definition (European Commission, 2011b); while the spICP-MS analysis would classify it as not being a nanomaterial. For the assessment of a nanomaterial, according to the EC definition, spICP-MS however qualifies as a powerful screening technique. In the case of a median diameter of below 100 nm, the material can be identified as a nanomaterial whereas in all other cases, spICP-MS needs to be combined with electron microscopy analysis as a confirmatory technique (Geiss et al., 2019; Verleysen et al., 2015).

The current legislation on food information to consumers (European Commission, 2011a) requires that ingredients presented in the form of engineered nanomaterials shall be clearly indicated in the list of ingredients followed by the word ‘nano’, without setting a threshold for the amount of nanoparticles. When testing the compliance of an ingredient material in relation to this legislation the spICP-MS method can, in most cases, provide sufficient evidence to confirm the need to label as a nanomaterial.

### 3.4 Comparison of ILC results with results obtained in other studies

Within the EU FP7-funded NanoDefine project (http://nanodefine.eu), a number of in-house validated methods went through interlaboratory studies. Single-particle ICP-MS was among the selected techniques, and the tested materials included a suspension of pristine coated (non-food grade) titanium dioxide powder and a sunscreen formulation containing titanium dioxide (NanoDefine, 2016). Although the tested materials were not the same, certain results obtained in the NanoDefine study can be compared with results of the current study (Table 9). The measurands evaluated in both studies include the mean particle diameter and the particle number concentration, and for both parameters, the repeatability and the reproducibility precision are considerably better in the study described in this work.

**Table 9:** Repeatability precision (RSD_r_) and reproducibility precision (RSD_R_) determined during the NanoDefine project and in this study for the mean particle diameter and the number concentration of titanium dioxide materials.

Measurand	Sample material	Study	RSD_r_	RSD_R_
Mean Particle Diameter	TiO_2_ in button-shaped candy	This study	2.0%	7.8%
	TiO_2_ in chewing Gum	This Study	3.9%	9.7%
	Pristine E 171	This Study	2.2%	11.8%
	TiO_2_ in suspension	NanoDefine	6.3%	43.0%
	TiO_2_ in sunscreen	NanoDefine	5.2%	39.0%
Particle Number Concentration	TiO_2_ in button-shaped candy	This study	9.2%	32.2%
	TiO_2_ in chewing Gum	This Study	13.1%	35.9%
	Pristine E 171	This Study	11.4%	43.1%
	TiO_2_ in suspension	NanoDefine	21.0%	97.0%
	TiO_2_ in sunscreen	NanoDefine	17.0%	79.0%

The improvement concerning the mean size may be explained by the different level of sample preparation complexity between the two studies. Due to different surface properties, the E 171 pristine material can be more easily dispersed than the material used in the NanoDefine study, and the extraction from sunscreen is likely to be more complex than the dissolution of sugar coatings.

## 4 Conclusions

This study proposes a method for the determination of the number-based particle size distribution and particle concentration of anatase, uncoated food-grade titanium dioxide in confectionery products. The method is based on single-particle ICP-MS as a screening method and transmission electron microscopy as a confirmatory technique. Given the broad diversity of existing food matrices in general but also within the category of confectionery, the development of a generally applicable method is challenging. Hence, this study specifically focused on confectioneries in which the titanium dioxide is dispersed in the outer sugar coating. Selecting such a relatively easy matrix allowed for a reduction in the uncertainty linked to the sample preparation step while covering numerous products on the market.

The transferability of this method was tested within an interlaboratory comparison study in which seven experienced European food control and food research laboratories participated. The overall results show a good repeatability and reproducibility for most measurands. A limitation of this study is the current lack of certified reference materials against which to assess the trueness of the results obtained using the method. Using transmission electron microscopy, which remains the golden standard for characterising and enumerating nanoparticles, as confirmatory technique, partly compensated for this limitation.

Although single-particle ICP-MS has a number of shortcomings such as relying on the assumption that particles have a near-spherical shape, not allowing for the direct determination of the particles’ minimum Feret diameter which is sometimes required in the legislative context, and not being able to discriminate between single constituent particles and constituent particles integrated in agglomerates/aggregates, it is nonetheless a powerful screening technique. It is a fast and easy to use technique which, in many cases, provides sufficient evidence to confirm the need to label a food product as containing (engineered) nanomaterial according to current EU regulatory requirements.

Inductively coupled plasma mass spectrometers are commonly available in laboratories doing routine analysis, and most of the more recent instruments can also be operated in single-particle mode. This is an important advantage over transmission electron microscopy.

The overall promising outcome of this work and at the same time the absence of alternative standardised procedures for the sizing and quantification of (engineered) nanoparticles in food matrices, make this method a candidate for a full validation study.

## Disclaimer

The information and views set out in this study are those of the author(s) and do not necessarily reflect the official opinion of the European Commission. The European Commission does not guarantee the accuracy of the data included in this study. Neither the European Commission nor any person acting on the European Commission's behalf may be held responsible for the use that may be made of the information contained therein.

## CRediT authorship contribution statement

**Otmar Geiss:** Methodology, Investigation, Data curation, Validation, Writing - original draft, Writing - review & editing, Visualization. **Ivana Bianchi:** Methodology, Investigation, Data curation, Validation, Writing - review & editing. **Chiara Senaldi:** Methodology, Investigation, Data curation, Validation, Writing - review & editing. **Guillaume Bucher:** Conceptualization, Writing - review & editing, Supervision, Investigation. **Eveline Verleysen:** Writing - review & editing, Supervision, Methodology, Data curation. **Nadia Waegeneers:** Writing - review & editing, Investigation, Data curation, Validation, Supervision. **Frédéric Brassinne:** Methodology, Investigation, Data curation, Validation. **Jan Mast:** Conceptualization, Writing - review & editing, Supervision. **Katrin Loeschner:** Conceptualization, Writing - review & editing, Supervision, Validation. **Janja Vidmar:** Writing - review & editing, Investigation, Data curation, Validation. **Federica Aureli:** Conceptualization, Writing - review & editing, Supervision, Validation. **Francesco Cubadda:** Conceptualization, Writing - review & editing, Supervision, Validation. **Andrea Raggi:** Methodology, Investigation, Data curation, Validation. **Francesca Iacoponi:** Methodology, Investigation, Data curation, Validation. **Ruud Peters:** Conceptualization, Writing - review & editing, Supervision. **Anna Undas:** Methodology, Investigation, Data curation, Validation. **Alexandra Müller:** Methodology, Investigation, Data curation, Validation. **Ann-Katrin Meinhardt:** Methodology, Investigation, Data curation, Validation. **Elke Walz:** Methodology, Validation, Writing - review & editing. **Volker Gräf:** Conceptualization, Writing - review & editing, Supervision. **Josefa Barrero-Moreno:** Project administration, Conceptualization, Writing - review & editing, Validation, Supervision.
