# Supplementary material for: Particle size analysis of pristine food-grade titanium dioxide and E 171 in confectionery products: Interlaboratory testing of a single-particle inductively coupled plasma mass spectrometry screening method and confirmation with transmission electron microscopy
Source: Food Control. 2021 Feb;120:107550. doi: 10.1016/j.foodcont.2020.107550 (PMC7730118; doi:10.1016/j.foodcont.2020.107550)

Supplementary Material (SM 1)

**Pictures of button shaped candies and chewing gum dragees used in this study.**


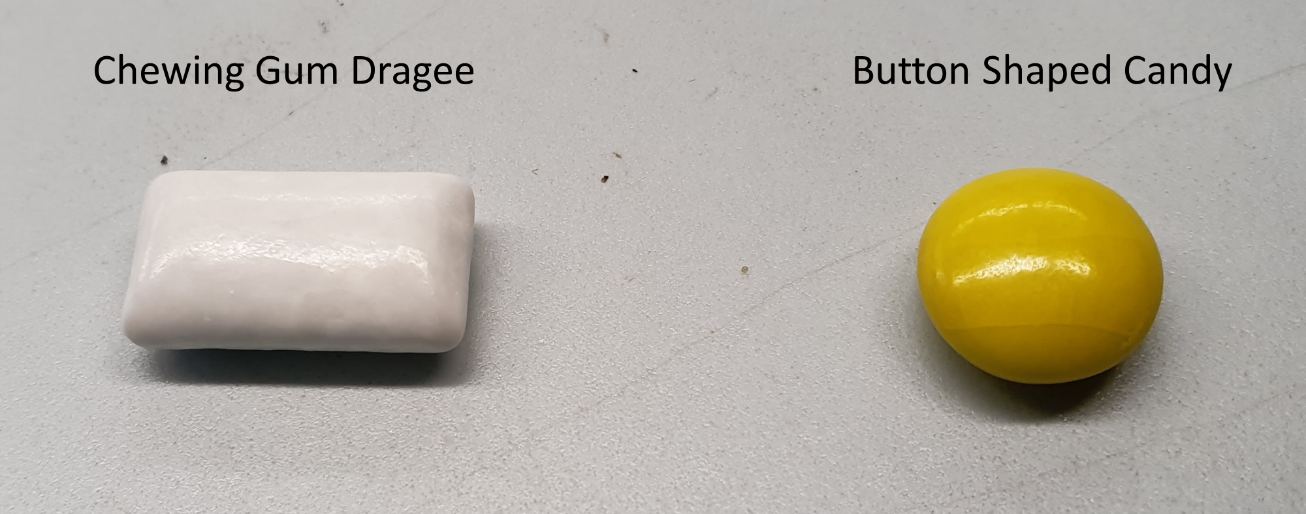

Supplement: Multimedia component 1 [file mmc1.docx]
