# Supplementary material for: Particle size analysis of pristine food-grade titanium dioxide and E 171 in confectionery products: Interlaboratory testing of a single-particle inductively coupled plasma mass spectrometry screening method and confirmation with transmission electron microscopy
Source: Food Control. 2021 Feb;120:107550. doi: 10.1016/j.foodcont.2020.107550 (PMC7730118; doi:10.1016/j.foodcont.2020.107550)
Supplement: Multimedia component 2 [file mmc2.docx]

Supplementary Material (SM2)

**Homogeneity testing of sample materials**

Sample homogeneity studies were conducted on the food samples using spICP-MS and on the pristine titanium dioxide using transmission electron microscopy (TEM).

**Homogeneity study on food samples with spICP-MS**

The homogeneity study conducted on the food samples was carried out following the procedures specified in ISO 13528:2015 (International Organization for Standardization, 2015). Ten units of chewing gum and ten units of button shaped candies were separately analysed in duplicate in random order under repeatability conditions. Samples were prepared according to the protocol specified under section 2.3 in the manuscript and analysed with spICP-MS. The following measurands were evaluated: mean particle diameter, most frequent particle diameter (mode), number concentration (i.e. number of particles per mL of suspension) and various number-weighted cumulative percentages (D10, D50, D99.5 and D99.8). The determination of homogeneity requires the calculation of the analytical precision (s_analytical_, repeatability standard deviation for one test portion, within sample) and the heterogeneity standard deviation (s_amples_, standard deviation across the test portions for one particular sample, between samples). Sample homogeneity was assessed by setting a target standard deviation (s_target_), against which the heterogeneity standard deviation of the samples (s_samples_) was compared. According to ISO 13528:2015, samples are considered as being homogeneous, if the heterogeneity standard deviation (s_sample_) does not exceed 30 % of the target standard deviation (s_target_). The recent study published by Bucher and Auger (Bucher & Auger, 2019) reports analytical precision values for the mean and the median size of E171 particles extracted from samples very similar to those investigated in this study. Intra-day and inter-day repeatability expressed as relative standard deviations were below 5 %. In the current study, the target standard deviation was set at double this value (10 %) for all measurands.

**Homogeneity study on pristine titanium dioxide with TEM**

The homogeneity study conducted on the pristine titanium dioxide powder was carried out following the procedures specified in the ‘General requirements for the competence of reference material producers’ (International Organization for Standardization, 2016). The number of units selected should correspond to approximately the cube root of the total of 300 units that were produced (in this case 7). Therefore, 10 units were selected using a random stratified sampling scheme covering the whole batch for the between-unit homogeneity test. For this, the batch was divided into 10 groups (with a similar number of units) based on the processing sequence, and one unit was selected randomly from each group. Duplicate measurements were conducted by means of TEM. The sample preparation consisted of weighing 88 mg of pristine E171 powder and bringing it in 35 mL of ultrapure water in a 50 mL polypropylene disposable tube. After 30 seconds of vortex stirring, an aliquot of 10 mL was transferred in a 20 mL glass vial. The sample was homogenously dispersed applying probe sonication under defined conditions (13 mm probe, 20% of amplitude, around 10 minutes sonication time) in order to deliver 10 kJ of acoustic energy. For details on how the effectively delivered energy was determined, please refer to section 2.3 of the manuscript. After sonication, 500 µL of the dispersion were transferred into 1.5 mL Eppendorf® caps and centrifuged at 6000 rpm (approximately 2000 g) for 2 hours. The supernatant was removed and the pellet was re-suspended in 500 µL of ultrapure water. About 15 µL of this dispersion were finally applied onto a pioloform- and carbon-coated 400 mesh copper grids (Agar Scientific, Essex, UK) which were previously pre-treated with Alcian blue. The grids were then left in contact with the suspension for 10 minutes. Hereafter, the grids were blotted to remove excess sample and air dried at room temperature. For each repetition, sample preparation of all units was performed on the same day and then the imaging of all units was performed on the next day. The image analysis was spread on seven days.

**Results**

**Homogeneity study on chewing gum and candies with spICP-MS and on pristine E171 with TEM**

Homogeneity is the acceptable distribution of chemical and physical properties within a batch, based on predefined criteria. The intent of examining homogeneity during the validation was to demonstrate that the quality of a sample collected from any location within a batch was representative of the quality of the entire batch. Table 1 includes the performance parameters used for the homogeneity assessment of the three tested materials. Homogeneity of chewing gum and the button shaped candies was determined with spICP-MS considering the mean particle diameter, the most frequent particle diameter (mode), a number of cumulative number/size percentages (D10, D50, D99.5, D99.8), the percentage of particles (in number) with a diameter smaller than 100 nm and the particles number concentration. The homogeneity of pristine E171 was tested with transmission electron microscopy. Measurands taken into consideration were the maximal Feret diameter (Fmax), the minimum Feret diameter (Fmin) and the particles aspect ratio, defined as Fmin/Fmax.

Table 1. Evaluation of samples homogeneity - Analysis with spICP-MS and with TEM

| Sample | Measurand | Unit | Mean | s(analytical)  [%] | s(sample)  [%] | s(target) for proficiency assessment [%] | ISO13528 – Test for adequate homogeneity |
| --- | --- | --- | --- | --- | --- | --- | --- |
| Chewing Gum  (spICP-MS) | Mean Particle Diameter | nm | 134 | 1.5 | 0.7 | 10 | Passed |
|  | Most Frequent Particle Diameter (Mode) | nm | 87 | 3.9 | 2.3 | 10 | Passed |
|  | D10 | nm | 64 | 2.1 | 0.4 | 10 | Passed |
|  | D50 | nm | 121 | 2.3 | 0.6 | 10 | Passed |
|  | D99.5 | nm | 332 | 5.9 | 3.3 | 10 | Passed |
|  | D99.8 | nm | 355 | 6.1 | 1.7 | 10 | Passed |
|  | Number of particles <100nm | % | 36.1 | 3.7 | 1.2 | 10 | Passed |
|  | Particle concentration | Particles  mL^-1^ | 76068 | 4.8 | 11.1 | 10 | Failed |
| Button shaped candies  (spICP-MS) | Mean Particle Diameter | nm | 140 | 1.7 | 1.4 | 10 | Passed |
|  | Most Frequent Particle Diameter (Mode) | nm | 91 | 4.3 | 1.3 | 10 | Passed |
|  | D10 | nm | 67 | 3.2 | 1.3 | 10 | Passed |
|  | D50 | nm | 126 | 2.3 | 1.6 | 10 | Passed |
|  | D99.5 | nm | 339 | 5.4 | 2.4 | 10 | Passed |
|  | D99.8 | nm | 367 | 4.3 | 3.3 | 10 | Passed |
|  | Number of particles <100nm | % | 33.7 | 5.9 | 1.7 | 10 | Passed |
|  | Particle concentration | Particles mL^-1^ | 52193 | 7.0 | 23.7 | 10 | Failed |
| E171  (TEM) | Fmax | nm | 109 | 3.7 | 0.3 | 10 | Passed |
|  | Fmin | nm | 91 | 3.7 | 1.5 | 10 | Passed |
|  | Aspect Ratio | - | 1.172 | 0.9 | 0.1 | 10 | Passed |

According to ISO 13528:2015, samples are considered as being homogeneous, if the heterogeneity standard deviation (s_sample_) does not exceed 30 % of the target standard deviation (s_target_). All test materials could be considered being adequately homogeneous according to ISO 13528:2015 for all measurands except the particles number concentration.

**References**

Bucher, G., & Auger, F. (2019). Combination of 47Ti and 48Ti for the determination of highly polydisperse TiO2 particle size distributions by spICP-MS. *Journal of Analytical Atomic Spectrometry*, *34*(7), 1380–1386. https://doi.org/10.1039/c9ja00101h

International Organization for Standardization. (2015). *Statistical methods for use in proficiency testing by interlaboratory comparison (ISO 13528:2015).*

International Organization for Standardization. (2016). *ISO 17034:2016. General requirements for the competence of reference material producers.*
