# Supplementary material for: Particle size analysis of pristine food-grade titanium dioxide and E 171 in confectionery products: Interlaboratory testing of a single-particle inductively coupled plasma mass spectrometry screening method and confirmation with transmission electron microscopy
Source: Food Control. 2021 Feb;120:107550. doi: 10.1016/j.foodcont.2020.107550 (PMC7730118; doi:10.1016/j.foodcont.2020.107550)
Supplement: Multimedia component 4 [file mmc4.docx]

Supplementary Material (SM4)

**Standard Operating Procedure for the Determination of the Particle Size Distribution (by number) of TiO_2_ Particles in Sugar Shell Confectionery (and pristine E171) by spICP-MS**

**Method:** Standard Operating Procedure for the Determination of the Particle Size Distribution (by number) of TiO_2_ Particles in Sugar Shell Confectionery (and pristine E171) by spICP-MS

**Analyte:** TiO_2_ (nano)particles determined as ^48^Ti

**Matrix:** Commercially available confectionery sugar shell products such as button shaped chocolate candies or dragee chewing gum

**Reference Document:**

ISO/TS 195590:2019 Nanotechnologies: Size distribution and concentration of inorganic particles in aqueous media via spICP-MS

*Version: 10/03/2020*

Contents

[Foreword 3](#_Toc19027958)

[1. Scope 3](#_Toc19027959)

[2. Terms and Definitions 4](#_Toc19027960)

[3. Theoretical background and summary of the analytical method 5](#_Toc19027961)

[4. Apparatus and Equipment 6](#_Toc19027962)

[5. Chemicals and Reagents 6](#_Toc19027963)

[6. Analysis 6](#_Toc19027964)

[6.1 Instrumental Settings 6](#_Toc19027965)

[6.2 Determination of the Transport efficiency (TE) (based on particle size) 7](#_Toc19027966)

[6.2.1 Sample flow measurement 7](#_Toc19027967)

[6.2.2 Preparation of Ionic Gold Calibration curve 7](#_Toc19027968)

[6.2.3 Preparation of NP Gold calibration curve 8](#_Toc19027969)

[6.3 Ionic calibration for the analysis of NP-TiO_2_ 9](#_Toc19027970)

[7. Sample preparation: m&m's and chewing gums 9](#_Toc19027971)

[7.1 Sampling and coating detachment 10](#_Toc19027972)

[7.2 Sonication of samples 10](#_Toc19027973)

[7.3 Dilution 10](#_Toc19027974)

[7.4 Preparation of blank 10](#_Toc19027975)

[7a. Sample preparation of Pristine E171 11](#_Toc19027976)

[8. Sample list and naming of samples 12](#_Toc19027977)

[9. Evaluation and reporting of results 13](#_Toc19027978)

Foreword

In 2018 the Joint Research Centre of the European Commission established a network with representatives of Member State (MS) control laboratories in charge of the enforcement of the legislative framework related to nanomaterials in food.

Aim of this network was to serve as an information exchange platform and to discuss the challenges and needs faced by the control laboratories for enforcement of the current legislative framework, in particular related to the ingredient labelling, and to identify the Joint Research Centre’s potential role/activities to support MS control laboratories.

Members of the network agreed to join forces towards progressing on method development/validation with focus on the determination of TiO_2_ in confectionery samples using sp-ICP-MS as screening method and TEM as confirmatory technique.

With the participation and assistance of the most experienced laboratories of the network, a standard operation procedure (SOP) for the measurement of titanium dioxide particles in confectionery was developed and tested in an interlaboratory study.

#

# 1. Scope

This Standard Operating Procedure (SOP) describes a screening procedure for the detection and determination of the number-size distribution of titanium-dioxide particles contained in the sugar-shell of confectionery (chewing gum and chocolate candies covered with a sugar shell) using single particle inductively coupled plasma mass spectrometry (sp-ICPMS). The SOP is strongly based on the working instruction developed by the French Service Commun des Laboratoires (SCL). The SOP can also be applied for the determination of the number size distribution of pristine E171.

# 2. Terms and definitions

spICP-MS Single Particle - Inductively Coupled Plasma Mass Spectrometry

NP Nano Particle. A particle with at least one dimension in the range of 1 to 100 nm.

TE Transport Efficiency

BK Blank

CG Chewing Gum

BS Bath Sonication

PS Probe Sonication

# 3. Theoretical background and summary of the analytical method

General principle of the spICP-MS method:

The basic principle of spICP-MS relies on the fact that nanoparticles can be detected individually if they are present in sufficiently diluted suspensions and the detector readout frequency (dwell time) is sufficiently high. Every single particle generates a discrete pulse of ions. The signal abundance is proportional to the mass of the particle. Particle size can then be calculated from the particle mass if an element-specific density and spherical geometry are assumed. The frequency of the detected signal pulses can be related to the particle number concentration in the suspension.

The analytical procedure consists of the following steps:

- Determination of sample flow rate of the peristaltic pump **(6.2.1)**
- Preparation of working standard for the determination of the transport efficiency (TE) and determination of the TE **(6.2.2 and 6.2.3)**
- Preparation of working standard for establishing the ionic titanium calibration curve **(6.3)**
- Sample preparation and dilution **(7)**

The samples are prepared by dispersion in ultrapure water and ultrasound sonication. The dispersion thus obtained is then diluted in ultra-pure water to reach a suitable particle concentration (recommended 1000-2000 particles/acquisition time).

OPTIONAL: for the analysis of pristine E171, follow instructions detailed under paragraph **7a**

- Analysis **(8)**
- Data processing according to the equipment software **(9)**

# 4. Apparatus and equipment

4.1 Analytical balance

4.2 Ultrapure water generation system

4.3 50 and 100 mL glass volumetric flasks

4.4 Variable or fix volumetric pipettes (10 µL, 100 µL, 1000 µL, 5000 µL and 10 mL)

4.5 Polypropylene disposable tubes (15 and 50 mL) with cap

4.6 Ultrasonic bath

4.7 Vortex mixer

4.8 Glass funnel

4.9 Inductively coupled plasma mass spectrometer composed of the following parts:

- Peristaltic pump
- Software with single particle application
- Glass nebulizer
- Glass cyclonic nebulization chamber
- Quartz torch
- PVC tube for sample uptake
- Santoprene tube for waste collection

4.10 Probe sonicator (for details see also section 7a.4)

# 5. Chemicals and reagents

5.1 Ultrapure water of quality 1 (NF EN ISO 3696), produced by the water purifier

5.2 Nitric acid (HNO_3_) 65-70%

5.3 Mono elemental solutions at 1000 mg L^-1^ (1000 ppm) of ionic titanium

5.4 Mono elemental solutions at 1000 mg L^-1^ (1000 ppm) of ionic gold

5.5 Monodisperse spherical gold nanoparticle (nanoComposix, 63 nm AuNP, coating citrate, 54 µg mL^-1^, 2.1*10^10^ particles mL^-1^)

# 6. Analysis

## 6.1 Instrumental settings

The following parameters are independent from the type of instrument used and are set as follows:

Ti (m/z) : 48

Density : 3.9 g mL^-1^ (Anatase)

Mass Fraction : 60%

Number of events (spikes) : 1000 – 2000

The following parameters need to be set according to the individual instrument characteristics:

RF Power (W)

Plasma Gas Flow (L min^-1^)

Aux Gas Flow (L min^-1^)

Neb Gas Flow (L min^-1^)

Peristaltic pump speed

Transport efficiency (for details see section 6.2)

Dwell-time [µS]

Settling-time (if applicable)

Total acquisition time

## 6.2 Determination of the transport efficiency (TE) (based on particle size)

### 6.2.1 Sample flow measurement

6.2.1.1 Determine the speed of the peristaltic pump.

6.2.1.2 Measure the suction flow rate of the pump by weighing difference with a tube filled with water over a period of at least 10 minutes.

6.2.1.3 Calculate the flow rate in mL min^-1^ considering the density of the water at 1 g mL^-1^

### 6.2.2 Preparation of ionic gold calibration curve

**Stock standard of ionic gold** (1 mg L^-1^ – 1 ppm)

6.2.2.1 Transfer 100 µL of 1000 ppm solution (5.4) into a 100 mL volumetric flask filled with approximately 50 mL of ultrapure water (5.1)

6.2.2.2 Bring to volume with ultrapure water (5.1). Mix thoroughly.

6.2.2.3 Label and store refrigerated (4 °C) at dark.

**Working standards of ionic gold** (0 – 10 µg L^-1^ or ppb)
According to the indications provided in table 1, pipet the volumes of the stock standard solutions (6.2.2.2) into 50 mL volumetric flask previously prefilled with approximately 20 mL ultrapure water and then bring to volume. Mix thoroughly.

The working standard needs to be prepared freshly each day of analysis.

Table 1. Working standards for ionic Gold

| **Concentration**  **[µg L^-1^]** | **Name** | **Dilution of Stock standard** | **Volume of Stock standard [µL]** | **Volume flask**  **[mL]** |
| --- | --- | --- | --- | --- |
| 0 (Blank) | Au^+^ Blank | - | 0 | 50 |
| 1 | Au^+^ Std 1 | 1:1000 | 50 | 50 |
| 3 | Au^+^ Std 2 | 1:333,33 | 150 | 50 |
| 5 | Au^+^ Std 3 | 1:200 | 250 | 50 |
| 10 | Au^+^ Std 4 | 1:100 | 500 | 50 |

### 6.2.3 Preparation of NP gold calibration curve

**Stock standard of 60 nm gold nanoparticles** (First dilution 1:100)

6.2.3.1 Let the monodisperse spherical gold nanoparticle suspension (5.5) reach room temperature and bath sonicate for 300s at the maximum power.

6.2.3.2 30 s vortex stirring of (6.2.3.1)

6.2.3.3 Pipet 500 µL of (6.2.3.1) in a 50 mL volumetric flask filled with approximately 20 mL of ultrapure water.

6.2.3.4 Bring to volume with ultrapure water (5.1). Mix thoroughly.

If not immediately used, label and store in refrigerator (4 °C) at dark.

If properly stored, the stock standard suspension can be considered stable for at least 15 days.

**Working standard of 60 nm gold nanoparticles** (Target concentration ca 100.000 particles mL^-1^]

*NOTE:* The reported dilution to 100000 particles mL^-1^ is used by way of example only and can be considered valid for instruments working at µs dwell time ranges. For ms dwell-time ranges, a higher dilution is required.

Pipette 50 µL of the stock standard (6.2.3.4) in a 100 mL volumetric flask filled with approximately 40 mL of ultrapure water (5.1) and bring to volume. This 1:2000 dilution results in a 105000 particles mL^-1^ suspension.

Vortex stir for 30 s

Transport Efficiency (TE) is either automatically calculated by the instrument's software or can be determined following the instructions provided in ISO/TS 19590 (Nanotechnologies – Size distribution and concentration of inorganic nanoparticles in aqueous media via single particle inductively coupled plasma mass spectromety).

## 6.3 Ionic calibration for the analysis of NP-TiO_2_

**Stock standard (A) of ionic titanium** (1 mg L^-1^ – 1 ppm=1000 ppb)

Assuming the supplied ionic Ti solution has a concentration of 1000 mg L^-1^, pipet 50 µL of 1000 ppm solution and 50 µL of HNO_3_ (68 %) into a 50 mL volumetric flask filled with approximately 20 mL of ultrapure water, then bring to volume with ultrapure water.

**Stock standard (B) of ionic titanium** (10 mg L^-1^ – 10 ppm=10000 ppb)

Assuming the supplied ionic Ti solution has a concentration of 1000 mg L^-1^, pipet 500 µL of 1000 ppm solution and 50 µL of HNO_3_ (68 %) into a 50 mL volumetric flask filled with approximately 20 mL of ultrapure water, then bring to volume with ultrapure water.

**Working standards of ionic titanium** (0 – 100 µg L^-1^ or ppb)
According to table 2 pipet the volumes of the stock standard solutions into 50 mL volumetric flasks previously filled with approximately 40 mL ultrapure water and then fill to the mark. Mix thoroughly.

Table 2. Working standards of ionic titanium

| **Concentration**  **[µg L^-1^]** | **Name** | **Volume of Ti^+^ Stock standard (A) [µL]** | **Volume of Ti^+^ Stock standard (B) [µL]** | **Volume HNO_3_[µL]** | **Volume flask [mL]** |
| --- | --- | --- | --- | --- | --- |
| 0 (Blank) | Ti^+^ Blank | 0 | - | 50 | 50 |
| 5 | Ti^+^ Std 2 | 250 | - | 50 | 50 |
| 10 | Ti^+^ Std 3 | 500 | - | 50 | 50 |
| 20 | Ti^+^ Std 4 | - | 100 | 50 | 50 |
| 50 | Ti^+^ Std 5 | - | 250 | 50 | 50 |

# 7. Sample preparation: button shaped candies and chewing gum

Samples are prepared and diluted on the same day of analysis. Samples should be analysed as soon as possible after sonication and dilution. If this is not possible, samples are kept at 4 °C before dilution on the day of analysis.

At all stages, it must be ensured that the level of contamination is as low as possible (clean glassware and plastic-ware according to good laboratory practice).

## 7.1 Sampling and coating detachment

For the sake of this exercise:

7.1.1 Weigh 3 chewing gum dragees or 6 sugar coated chocolate candies into a 50 mL polypropylene disposable tube and note the weight (approx. 6 g).

7.1.2 Add 25 mL of ultrapure water (5.1) by means of a volumetric pipette (4.4)

7.1.3 Manually stir/shake samples until the coating is entirely removed (chocolate core of sugar coated chocolate candies and grey gum mass of chewing gum dragee become visible, without any residual white material attached).

7.1.4 Pour all the content of the polypropylene tube through a funnel collecting the suspension in a new 50 mL polypropylene disposable tube.

7.1.5 Rinse the first polypropylene tube with 10 mL of ultrapure water (5.1) and use it to rinse the candies (or the chewing gums) which remained in the funnel.

Total Volume: 35 mL

**NB:** Apply this procedure to each of the replicates (3 tubes containing 3 chewing gum dragees + 3 tubes containing 6 chocolate candies)

## 7.2 Sonication of samples

*(Blank samples (see 7.4) can be prepared in parallel)*

7.2.1 Bath sonicate the dispersions at the maximum power for 600s (10 min).

7.2.2 Vortex stir for 30s

Measure the pH value by means of pH paper (it should be in the range 5.5-7.0).
If the pH value is lower, please adjust adding dropwise diluted sodium hydroxide solution.

## 7.3 Dilution

Dilution is done in such a way that the number of particles ranges from 1000 to 2000 per acquisition time.

1 to 100000 dilution (D100000) is reported hereafter as an example

7.3.1 First dilution D1000

Pipette 50 µL of 7.2.4 to 50 mL of ultrapure water (5.1)

7.3.2 Second dilution D100

Pipette 500 µL of 7.3.1 to 50 mL of ultrapure water (5.1)

## 7.4 Preparation of blank

This represents a negative control for excluding any possible contamination.

For its preparation it is necessary to replicate all the steps taken for the preparation of the samples.

Precisely:

7.4.1 Add 25 mL of ultrapure water (5.1) to a 50 mL polypropylene disposable tube by
 means of a volumetric pipette (4.4)

7.4.2 Pour all the content of the polypropylene tube through a funnel collecting the ultrapure water in a new 50 mL polypropylene disposable tube.

7.4.3 Rinse the first polypropylene tube with 10 mL of ultrapure water (5.1) and pour them into the second tube through the funnel.

7.4.4 Bath sonicate at the maximum power for 600s (10 mins)

7.4.5 Vortex stirring for 30s

7.4.6 Apply the same dilution factor as for the "real" sample.

**NB:** Prepare one blank for each day of analysis (in case samples are not analysed on the same day).

*****OPTIONAL**

# 7a. Sample preparation of pristine E171

*(Blank samples can be prepared in parallel)*

7a.1 Weigh 40 mg of pristine E171 in a 50 mL polypropylene disposable tube and add 40 mL of ultrapure water (5.1) – (1 mg mL^-1^)

7a.2 Vortex stirring for 30s

7a.3 Transfer 10 mL of this suspension (7a.2) into a 15 mL polypropylene disposable tube

NOTE: Transfer could lead to the loss of sample and direct sonication is preferable IF available tip diameter is appropriate for the volume of suspension to be sonicated.

7a.4 Probe sonicate the dispersion (7a.3) (delivered energy 10KJ). Cool down the sample during sonication, by immersion in ice/water bath.

NOTE: As the sonication probe tip might heat up during the sonication, let the it cool down after each sonication step.

Power output characteristics of the deployed sonication system need to be determined following the protocol published in the NanoDefine Technical Report D2.3^[[1]](#footnote-1)^. The relevant extract of this report is annexed to this SOP.

Once the effective power output has been exactly determined (ideally around 18 W), the necessary sonication time can be calculated according to the following formula:

Time [min] = 10000 J / (power output [W] * 60 s)

N.B. If an appropriate probe sonicator is being used, resulting time should not deviate much from 10min.

7a.6 Vortex stirring for 30s

7a.7 Dilute with ultrapure water (5.1) so that the number of particle ranges from 1000 to 2000 within the acquisition time (scan time).

**Preparation of pristine E171 blank**

7a.8 Add 40 mL of ultrapure water (5.1) by means of a volumetric pipette (4.4)

7a.9 Transfer 10 mL of this suspension (7a.10) into a 15 mL polypropylene disposable tube

7a.10 Probe sonicate the dispersion (7a.11) (delivered energy 10KJ). Cool down the sample during sonication, by immersion in ice/water bath.

7a.11 Vortex stirring for 30s

7a.12 Apply same dilution factor as for the "real" samples.

**NB:** Prepare one blank for each day of analysis (in case samples are not analysed on the same day).

# 8. Sample list and naming of samples

Ionic Ti calibration is included at the beginning and at the end of the sequence to verify instrument performance over the course of the run.

A blank with ultrapure water is included before and after samples set analysis to check for memory effects.

In order to verify the stability of measurement, please measure three consecutive injections for each sample (leave sampling tube in sample and re-analyse same sample).

A typical sample sequence looks like as follows:

Determination of Transport Efficiency

Au^+^ Blank (MilliQ Water)

Au^+^ Std 1

Au^+^ Std 2

Au^+^ Std 3

Au^+^ Std 4

Np-Au Blank (MilliQ Water)

Np-Au 60nm std 100000 particles mL^-1^

Ti^+^ Blank

Ti^+^ Std 1

Ti^+^ Std 2

Ti^+^ Std 3

TI^+^ Std 4

TI^+^ Std 5

Flush the instrument for 10 min with MilliQ Water

MilliQ Water (as sample)

BK_m&m's_BS_dxxx*

m&m's_BS_1_dxxx

m&m's_BS_2_dxxx

m&m's_BS_3_dxxx

MilliQ Water (as sample)

BK_CG_BS_dxxx

CG_BS_1_dxxx

CG_BS_2_dxxx

CG_BS_3_dxxx

MilliQ Water (as sample)

BK_E171_PS_dxxx

Optional

E171_1_PS_dxxx

E171_2_PS_dxxx

E171_3_PS_dxxx

MilliQ Water (as sample)

Ti^+^ Blank

Ti^+^ Std 1

Ti^+^ Std 2

Ti^+^ Std 3

TI^+^ Std 4

TI^+^ Std 5

*= sample final dilution

# 9. Evaluation and reporting of results

The raw data are processed with the instrument’s software. Please note that some of the required parameters might not be automatically calculated by the single particle module of the ICP software and need therefore to be generated with external data treatment software packages.

Parameters

- Instrument parameters
  - Transport efficiency (%);
  - Peristaltic pump sample flow rate (mL min^-1^)
- Most frequent particle diameter – MODE (nm)

The most frequent size can be determined in various ways.

- - The preferred way is to determine the most frequent size through the Kernel Density Estimate. For this approach the data needs to be exported and to be elaborated with data elaboration software packages such as Origin. A detailed procedure can be found in annex 1.
  - If the determination through the Kernel Density Estimate is not possible, the most frequent size should be extracted from a histogram setting the bin size at 10 nm.
  - If neither the Kernel Density approach nor the 10nm bin-size approach is possible, please indicate how it was determined (e.g. through histogram with 2 nm bin-size).
- Size range (Minimum and maximum) of detected particles
  - Lower end of size range: the lower end of the size range can coincide with the lowest detectable size which depends on the daily instrument performance and which can therefore slightly vary on different days of measurement.
  - Upper end of the size range: To exclude isolated very large agglomerates/aggregates, the upper end of the size range is determined as the D99,5 value^[[2]](#footnote-2)^.
- Particle mean diameter (nm)
- Median - Diameter at which 50% of the sample's particles are comprised of particles with a diameter less than this value (D50, nm)
- Diameter at which 10% of the sample's particles are comprised of particles with a diameter less than this value (D10, nm)
- Percentage of particles having diameter smaller than 100 nm
- Number of particles *per* scan time (n particles min^-1^)

**Annex 1. Determination of 'Most Frequent Size' through the Kernel Density Estimation using OriginPro 2015 for elaboration of data exported from Perkin Elmer Syngistix V1.1.**

a) Open Origin Pro and copy size in column a and frequency for each size in column B


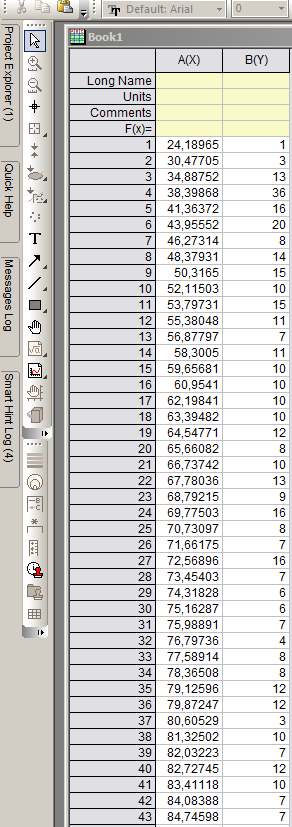


b) Add empty column (Column C) – Right mouse click on space where third column should be added and select "Add New Column".


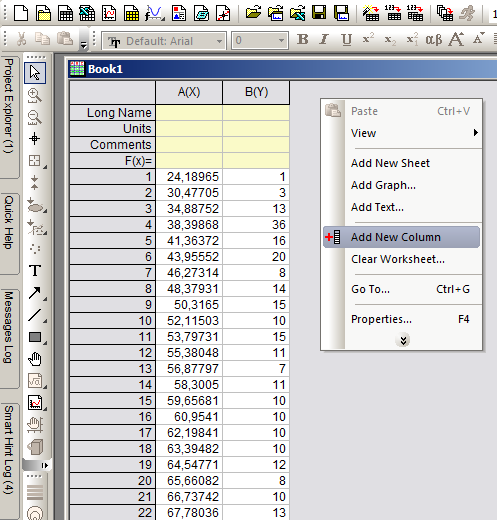


c) Open Window-Script Window from pull-down menu


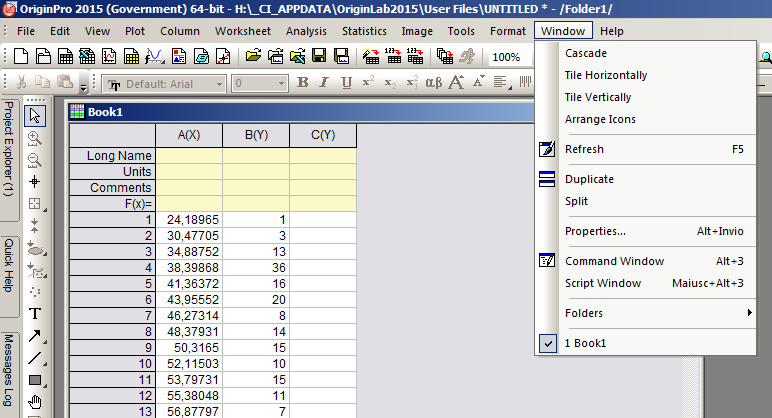


d) Type the following code into the Script Window and highlight (select) the text

function dataset CreateData(dataset ds1, dataset ds2)

{

dataset ds3;

ds3.SetSize(total(ds2));

int idx = 1;

for (int i = 1; i <= ds2.GetSize(); i++)

{

for(int j=1; j <= ds2[i]; j++)

{

ds3[idx]=ds1[i];

idx++;

}

}

return ds3;

}

col(C) = CreateData(col(A),col(B));


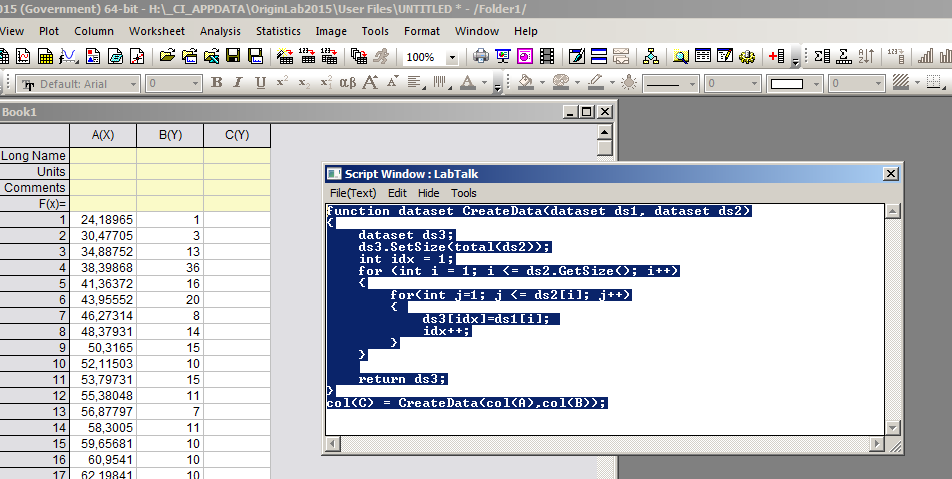


e) Press <Enter>

f) Now column C should contain all individual values


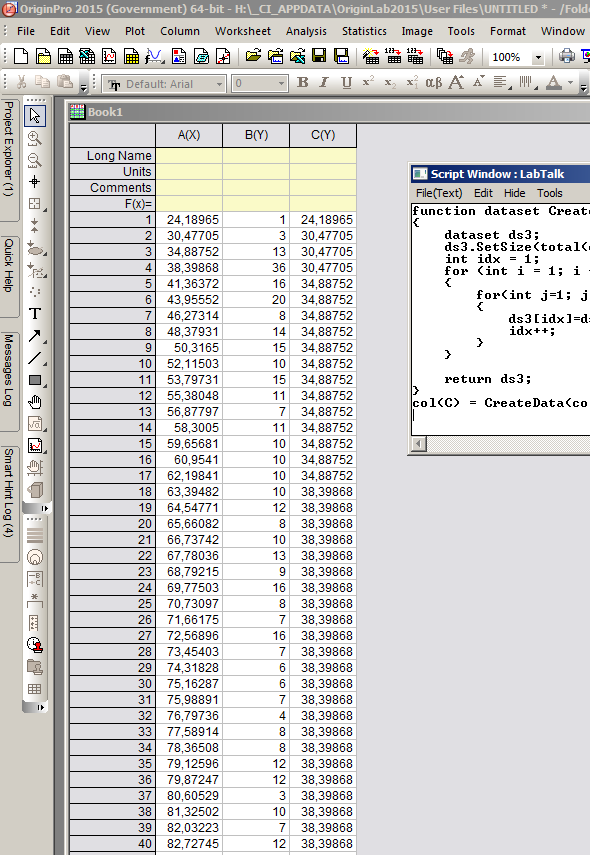


g) Close Script Window and select column C

h) Go in PLOT-STATISTICS_HISTOGRAM

i) Double click left mouse button and in DISTRIBUTION CURVE select KERNEL SMOOTHING


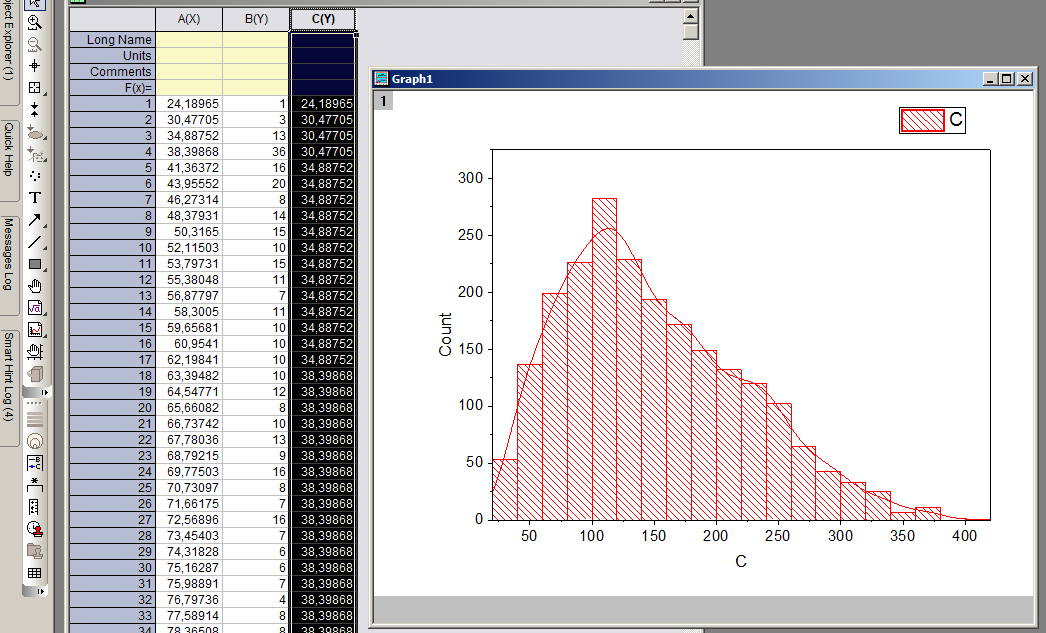


1. The full technical report can be downloaded at http://www.nanodefine.eu/publications/reports/NanoDefine_TechnicalReport_D2.3.pdf [↑](#footnote-ref-1)
2. Diameter at which 99.5% of the sample's particles are comprised of particles with a diameter less than this value. [↑](#footnote-ref-2)
