# Supplementary material for: Particle size analysis of pristine food-grade titanium dioxide and E 171 in confectionery products: Interlaboratory testing of a single-particle inductively coupled plasma mass spectrometry screening method and confirmation with transmission electron microscopy
Source: Food Control. 2021 Feb;120:107550. doi: 10.1016/j.foodcont.2020.107550 (PMC7730118; doi:10.1016/j.foodcont.2020.107550)

Supplementary Material (SM5)

**TEM micrographs (a,c,e) and corresponding annotated micrographs (b,d,f) resulting from image analysis by ellipse fitting of pristine E171 (a, b), button shaped candies (c,d) and chewing gum (e,f). Particle detection and measurement is indicated on the annotated micrographs by the red ellipses.**

**(a)**


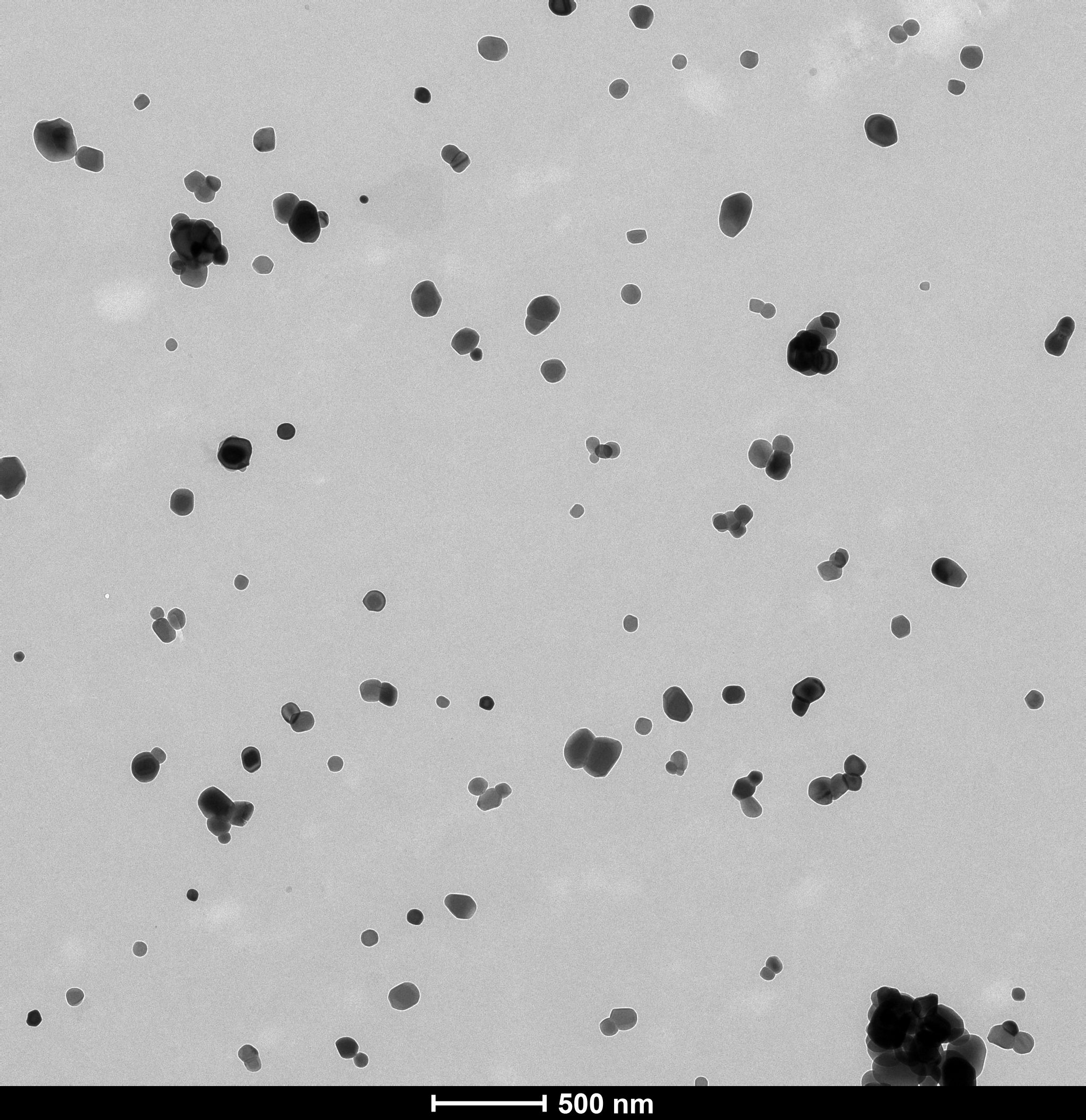


**(b)**


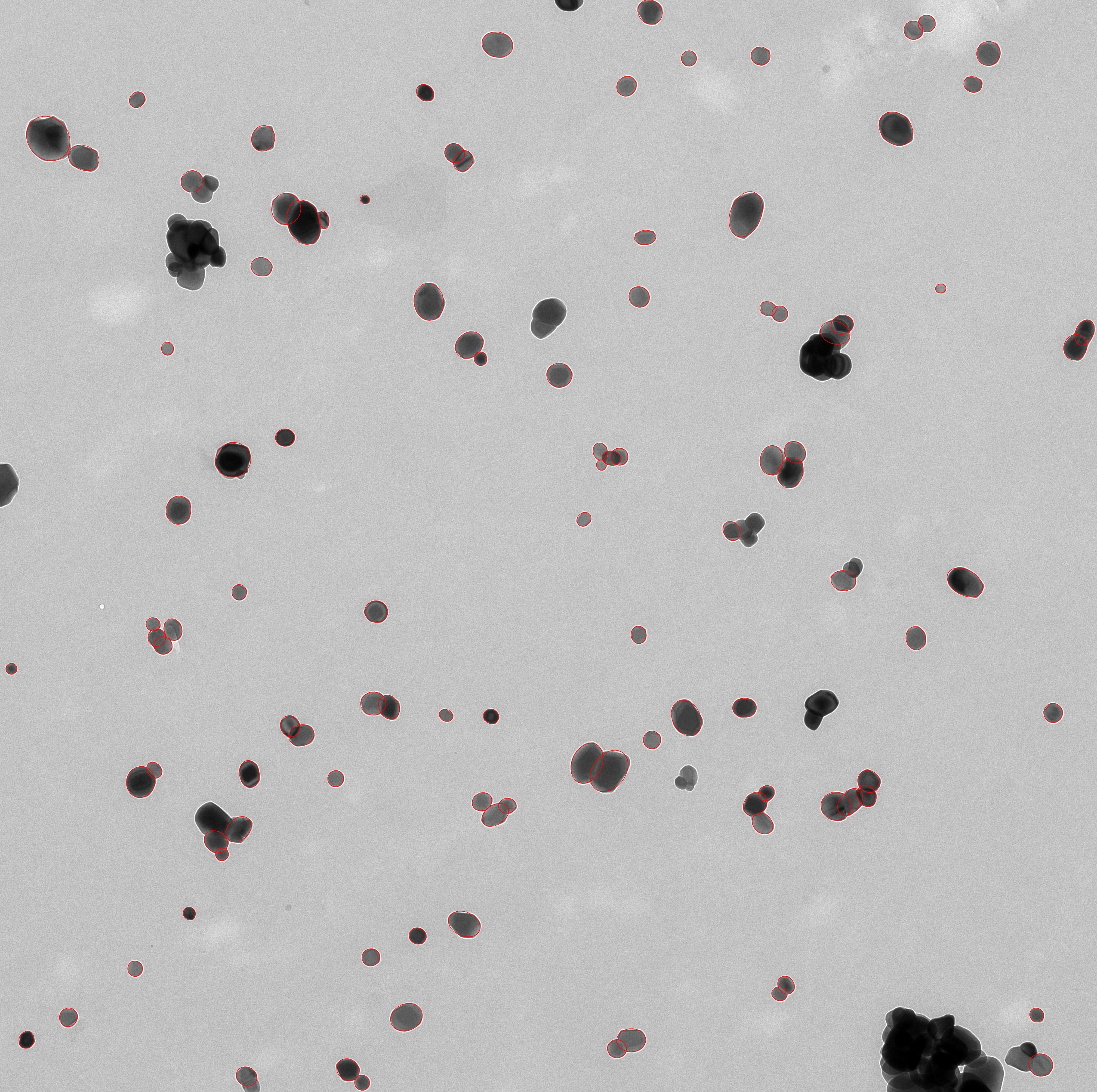


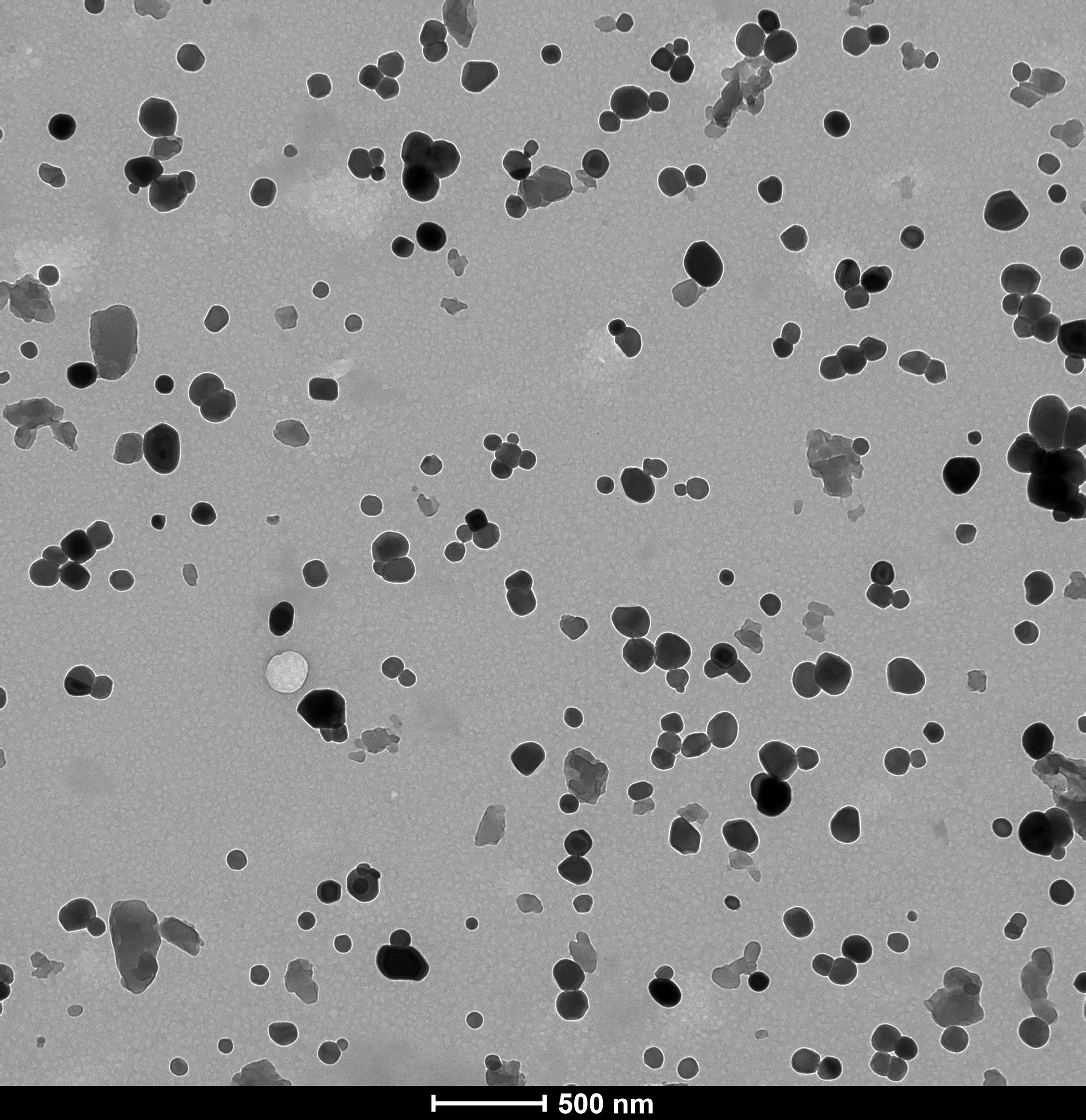

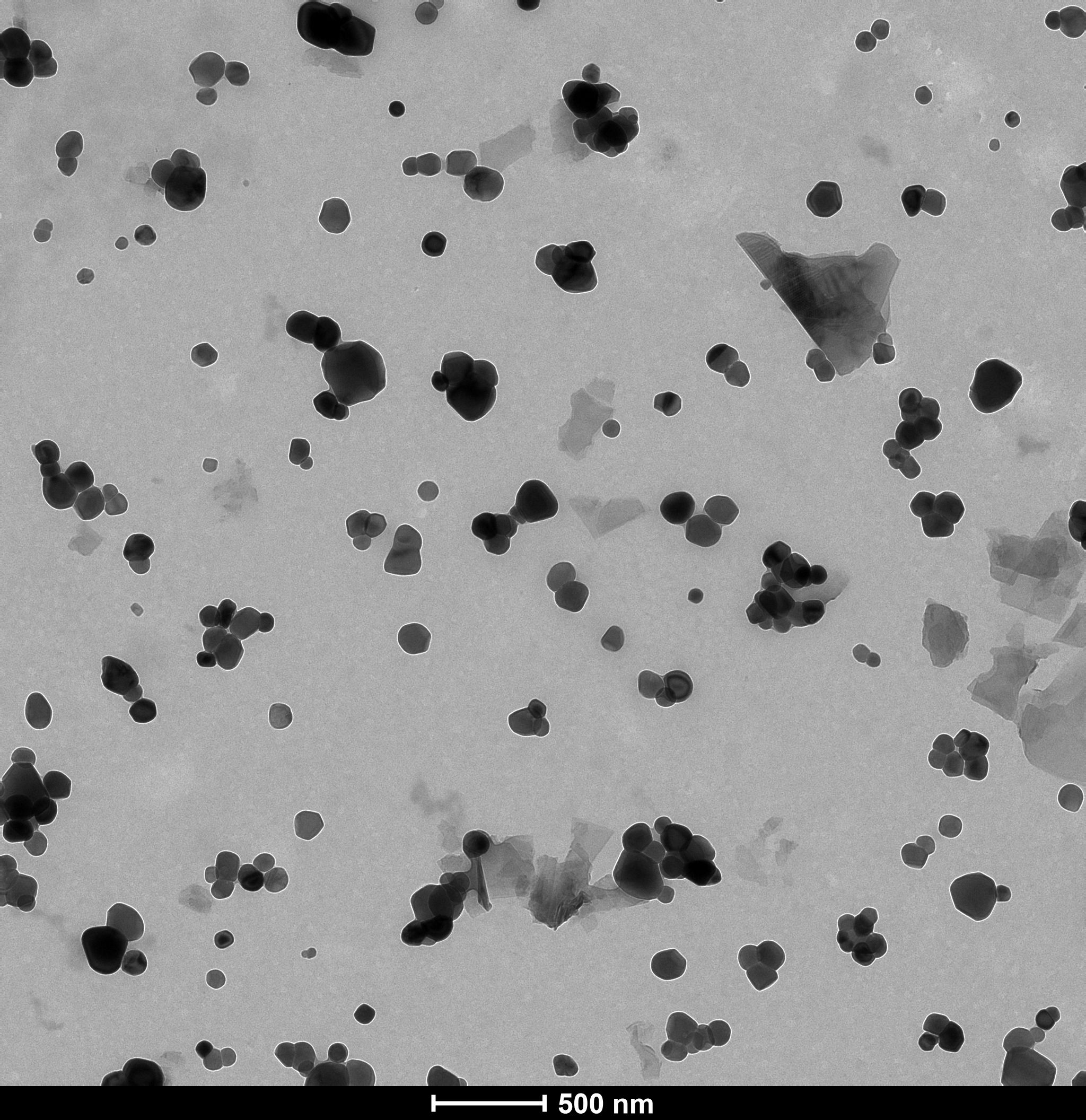


**(c)**

**(d)**


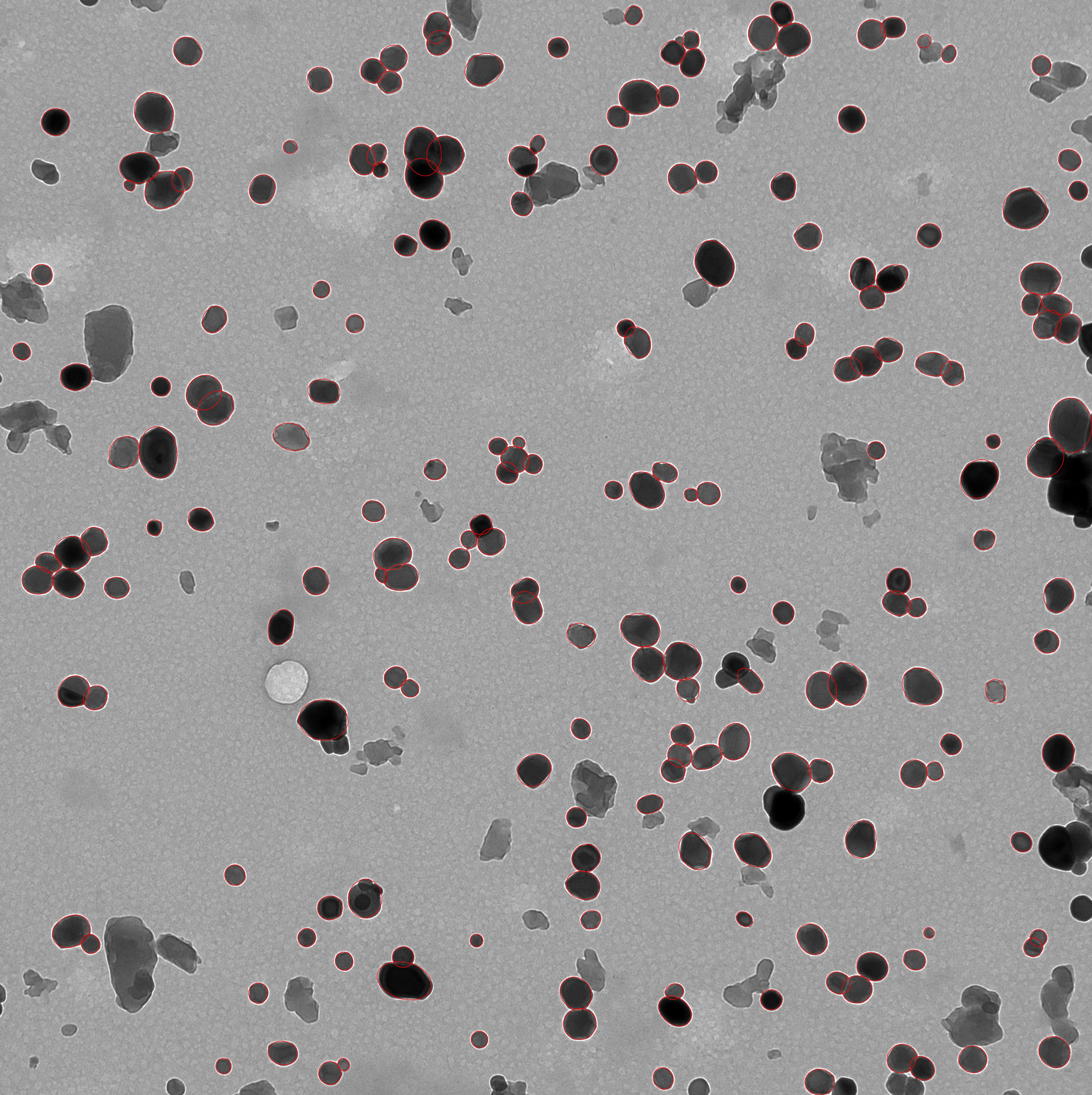


**(e)**

**(f)**


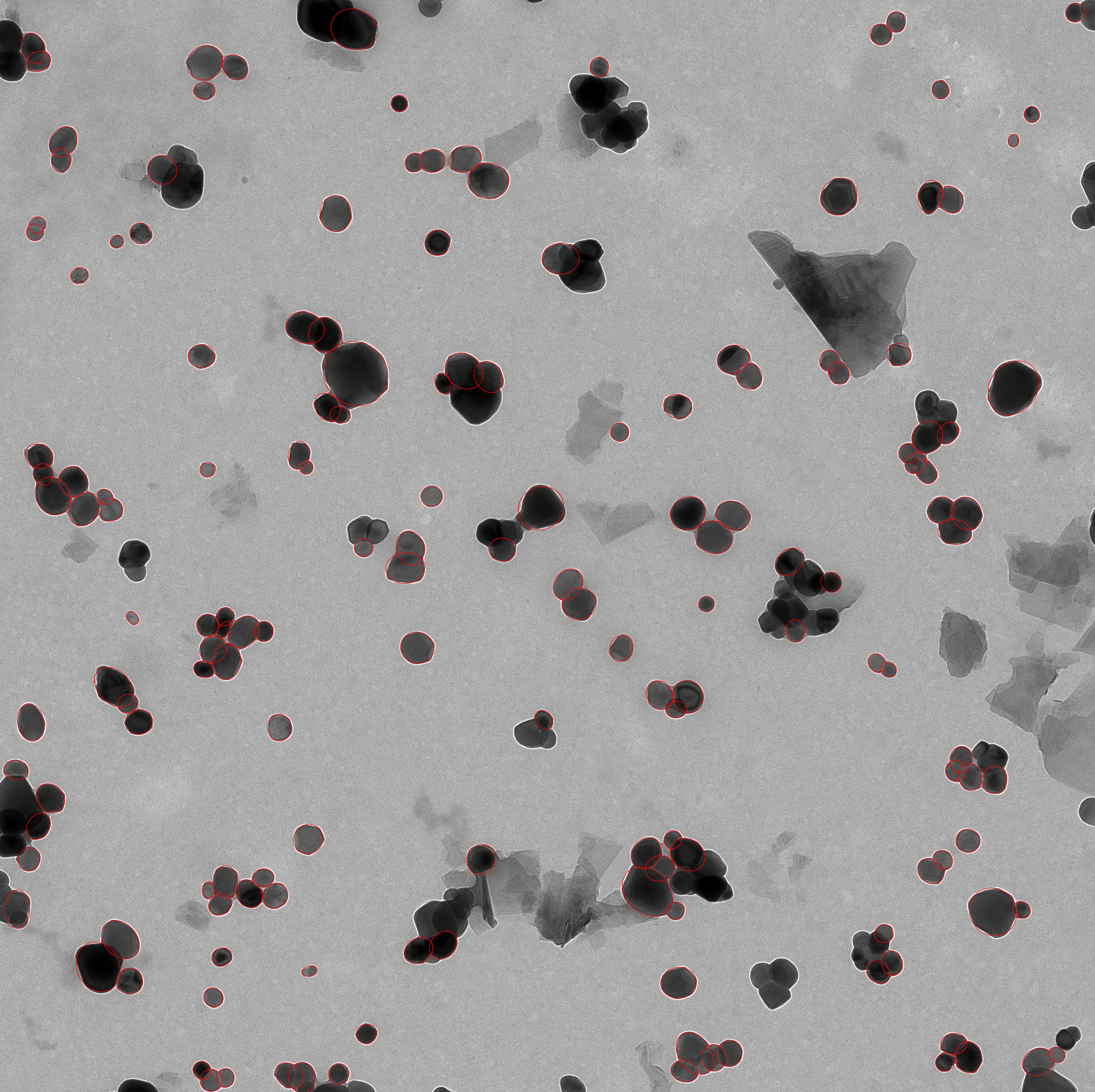

Supplement: Multimedia component 5 [file mmc5.docx]
